# Doping-driven topological polaritons in graphene/α-MoO_3_ heterostructures

**DOI:** 10.1038/s41565-022-01185-2

**Published:** 2022-08-18

**Authors:** Hai Hu, Na Chen, Hanchao Teng, Renwen Yu, Yunpeng Qu, Jianzhe Sun, Mengfei Xue, Debo Hu, Bin Wu, Chi Li, Jianing Chen, Mengkun Liu, Zhipei Sun, Yunqi Liu, Peining Li, Shanhui Fan, F. Javier García de Abajo, Qing Dai

**Affiliations:** 1 CAS Key Laboratory of Nanophotonic Materials and Devices, CAS Key Laboratory of Standardization and Measurement for Nanotechnology, CAS Center for Excellence in Nanoscience, National Center for Nanoscience and Technology, Beijing, People’s Republic of China; 2 University of Chinese Academy of Sciences, Beijing, People’s Republic of China; 3 ICFO-Institut de Ciencies Fotoniques, The Barcelona Institute of Science and Technology, Castelldefels, Spain; 4 Department of Electrical Engineering, Ginzton Laboratory, Stanford University, Stanford, CA USA; 5 Beijing National Laboratory for Molecular Sciences, Key Laboratory of Organic Solids, Institute of Chemistry, Beijing, People’s Republic of China; 6 The Institute of Physics, Chinese Academy of Sciences, Beijing, People’s Republic of China; 7 Department of Physics and Astronomy, Stony Brook University, NY, USA; 8 Department of Electronics and Nanoengineering, Aalto University, Espoo, Finland; 9 Wuhan National Laboratory for Optoelectronics and School of Optical and Electronic Information, Huazhong University of Science and Technology, Wuhan, People’s Republic of China; 10 ICREA-Institució Catalana de Recerca i Estudis Avançats, Barcelona, Spain

**Keywords:** Nanophotonics and plasmonics, Nanophotonics and plasmonics, Polaritons

## Abstract

Control over charge carrier density provides an efficient way to trigger phase transitions and modulate the optoelectronic properties of materials. This approach can also be used to induce topological transitions in the optical response of photonic systems. Here we report a topological transition in the isofrequency dispersion contours of hybrid polaritons supported by a two-dimensional heterostructure consisting of graphene and α-phase molybdenum trioxide. By chemically changing the doping level of graphene, we observed that the topology of polariton isofrequency surfaces transforms from open to closed shapes as a result of doping-dependent polariton hybridization. Moreover, when the substrate was changed, the dispersion contour became dominated by flat profiles at the topological transition, thus supporting tunable diffractionless polariton propagation and providing local control over the optical contour topology. We achieved subwavelength focusing of polaritons down to 4.8% of the free-space light wavelength by using a 1.5-μm-wide silica substrate as an in-plane lens. Our findings could lead to on-chip applications in nanoimaging, optical sensing and manipulation of energy transfer at the nanoscale.

## Abstract (web summary)

Polaritonic topological transitions of the isofrequency dispersion contour are observed in a graphene/α-MoO_3_ heterostructure by tuning the graphene doping level, which enables partial focusing at deep subwavelength.

## Main

The control of charge carrier concentration by either electrostatic or chemical means has been widely studied as a way to induce phase transitions of different nature, such as structural in transition metal dichalcogenides^1–4^, ferromagnetic in high-Curie-temperature manganites^5–10^ and topological in engineered materials^11–14^, with potential application in the development of active electronic phase-change devices^15^. In this context, a collection of different phases in magic-angle bilayer graphene has been achieved by changing its carrier density^16^. Similar concepts have been theoretically explored in photonics using hyperbolic metamaterials composed of subwavelength structures, such as a periodic array of graphene ribbons^17^ or a stack of graphene dielectric layers^18^, in which a topological transition in the isofrequency dispersion contour can occur by changing the doping level of graphene. However, these hyperbolic metamaterials rely on a strong anisotropy of the effective permittivity tensor, which is ultimately limited by spatial nonlocal effects that can hinder a practical verification of this concept.

Recently, a twisted stack of two α-phase molybdenum trioxide (α-MoO_3_) films was explored to control the topology of the isofrequency dispersion contour of phonon polaritons (PhPs) by varying the relative twist angle between the two α-MoO_3_ layers^19–22^. Owing to the in-plane anisotropy of the permittivity within the reststrahlen band from 816 to 976 cm^−1^, the real part of the permittivity is positive along the [001] direction but negative along the [100] direction^23,24^, a property that renders α-MoO_3_ a natural hyperbolic material supporting in-plane hyperbolic PhPs^25,26^. The low-loss in-plane hyperbolic PhPs in α-MoO_3_ thus emerge as an ideal platform to explore further possibilities of doping-driven and electrically tunable topological transitions in photonics.

In this work we have achieved the control of polariton dispersion in a van der Waals (vdW) heterostructure composed of an α-MoO_3_ film covered with monolayer graphene by changing the doping level of the latter. We observed the polariton dispersion contour to vary from hyperbolic (open) to elliptic (closed) on increasing the doping level of graphene, leading to the emergence of a mode dominated by its graphene plasmon polariton (GPP) component propagating along the [001] direction at high doping levels. The nature of the polaritons emerging at high doping in the heterostructure evolved from GPP to PhP when moving from the [001] to [100] α-MoO_3_ crystallographic direction. In addition, when the vdW heterostructure was placed on top of a gold substrate instead of SiO_2_, a rather flattened dispersion contour was obtained due to a topological transition. As an application, we have designed an in-plane subwavelength focusing device by engineering the substrate.

## Tunable topological polaritons in heterostructures

**Fig. 1:**
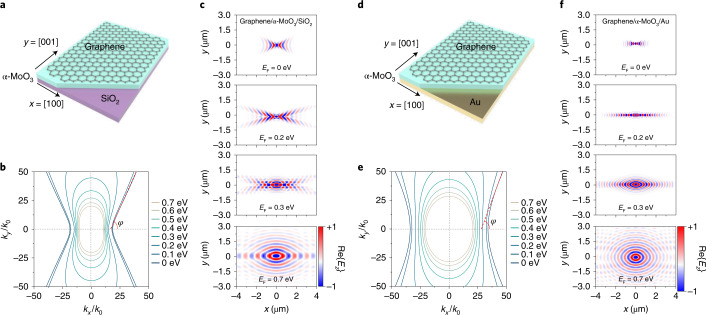
Topological transition of hybrid polaritons. **a**,**d**, Illustration of the graphene/α-MoO_3_ vdW heterostructure used in this study, supported on SiO_2_ (**a**) and gold (**d**) substrates. **b**,**e**, Calculated isofrequency dispersion contours of hybrid polaritons on a 300-nm-thick SiO_2_ substrate (**b**) and a 60-nm-thick gold substrate (**e**) at a fixed incident frequency of 910 cm^−1^ (*λ*_0_ = 10.99 µm) for different graphene Fermi energies ranging from 0 to 0.7 eV and an α-MoO_3_ film thickness of 150 nm. *φ* indicates the opening angle of the hyperbolic sectors. *k*_x_ and *k*_y_ are the momenta of polariton along the *x* and *y* crystal directions of α-MoO_3_, while *k*_0_ is the momentum of light in free space. **c**,**f**, Numerically simulated field distributions (real part of the *z* out-of-plane component of the electric field, Re{*E*_*z*_}) of hybrid polaritons on SiO_2_ (**c**) and gold (**f**) substrates for several graphene doping levels at a fixed incident frequency of 910 cm^−1^, as launched by a dipole placed 100 nm above the origin.

A schematic of our proposed structure is shown in Fig. 1, where a 150-nm-thick vdW heterostructure is placed on top of either a SiO_2_ (Fig. 1a) or gold (Fig. 1d) substrate. We were particularly interested in the reststrahlen band II of α-MoO_3_ extending over the frequency range of 816 to 976 cm^−1^, where the permittivity (*ε*) components along the [100], [001] and [010] crystal directions satisfy *ε*_*x*_ < 0, *ε*_*y*_ > 0 and *ε*_*z*_ > 0, respectively (Supplementary Fig. 1a)^23,24^. As a result, the in-plane PhPs in natural α-MoO_3_ exhibit a hyperbolic dispersion contour. To illustrate dynamic control of the dispersion contour topology of the in-plane hybrid plasmon–phonon polaritons in our structure, we present in Fig. 1b several calculated dispersion contours on SiO_2_ at different graphene Fermi energies (*E*_F_) under a fixed representative incident free-space wavelength of *λ*_0_ = 10.99 μm (frequency of 910 cm^−1^). As the graphene Fermi energy increases from 0 to 0.7 eV, the opening angle *φ* of the hyperbolic sectors gradually increases due to a change in the PhP wavelength when varying the dielectric environment, and eventually the dispersion contour changes its character from a hyperbolic (open) to elliptic (closed) shape. Note that the Fermi energy at which this topological transition occurs is conditioned to the appearance of well-defined graphene plasmons along the [001] direction at *λ*_0_ when increasing its doping level^27–30^. When the substrate was changed from SiO_2_ to gold, we found flatter dispersion contours (Fig. 1e) due to the stronger effect of screening provided by the gold substrate. Tunable polariton canalization and diffractionless propagation were thus expected.

Numerically simulated field distributions of hybrid polaritons for different graphene Fermi energies are shown in Fig. 1c,f. At *E*_F_ = 0 eV, the polaritons of the heterostructure on a SiO_2_ substrate exhibit a hyperbolic wavefront, similar to that of the PhPs in α-MoO_3_. In contrast, the wavelength of the polaritons on a gold substrate is highly compressed, whereas their opening angle is increased. On increasing the doping level to *E*_F_ = 0.2 eV, a topological transition takes place. The wavelength of the hybrid polaritons is increased compared with the undoped case, and we can still observe a hyperbolic wavefront along the *x* direction on the SiO_2_ substrate. Moreover, for the hybrid polaritons on the gold substrate (Fig. 1f), highly collimated and directive hybrid polaritons propagating along the *x* direction can be observed as a result of a rather flattened dispersion contour. At *E*_F_ = 0.3 eV, a hyperbolic wavefront of hybrid polaritons can still be observed along the *x* direction with the SiO_2_ substrate, but another mode with an elliptic wavefront propagating along the *y* direction also emerges. With the gold substrate, the wavefront of the hybrid polaritons is dominated by a fine crescent shape along the *x* direction. At a higher doping level (*E*_F_ = 0.7 eV), the dispersion contours for the hybrid polaritons on both SiO_2_ and gold substrates display an elliptic-like shape (Fig. 1b,e). As a consequence, we can find modes propagating anisotropically in the *x*–*y* plane (for additional theoretical analyses, see Supplementary Figs. 2–4 and other works^31–33^).

## Experimental observation of topological transitions

**Fig. 2:**
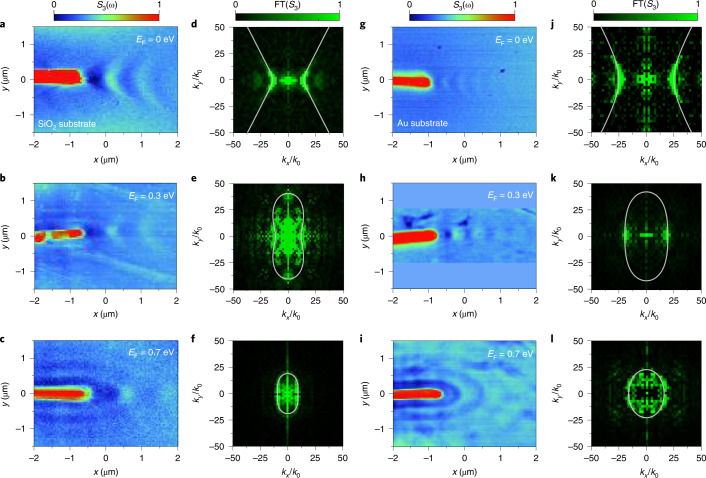
Topological transition of hybrid polaritons revealed by nanoimaging. **a**–**c**, Experimentally measured polariton near-field amplitude (*S*_3_) images with graphene doping *E*_F_ = 0 eV (**a**), *E*_F_ = 0.3 eV (**b**) and *E*_F_ = 0.7 eV (**c**). The polaritons were launched by a gold antenna. The α-MoO_3_ film was placed on top of a 300 nm SiO_2_/500 μm Si substrate. **d**–**f**, Absolute value of the spatial Fourier transforms (FTs) of the experimental near-field images shown in **a**–**c**, respectively, revealing the isofrequency contours of hybrid polaritons. The grey curves represent calculated isofrequency contours. **g**–**i**, Experimentally measured polariton near-field amplitude (*S*_3_) images with graphene doping *E*_F_ = 0 eV (**g**), *E*_F_ = 0.3 eV (**h**) and *E*_F_ = 0.7 eV (**i**) for an α-MoO_3_ film placed on a 60 nm Au/500 μm Si substrate. The canalized wavefronts were measured at a graphene Fermi energy close to the value at which the topological transition occurs (**h**), showing deep-subwavelength and diffractionless polariton propagation. **j**–**l**, Absolute value of the FTs of the experimental near-field images in **g**–**i**, respectively. The grey curves show the calculated isofrequency contours. The α-MoO_3_ thickness was 140 nm in all panels. The incident light wavelength was fixed at *λ*_0_ = 11.11 μm (900 cm^−1^). Each colour scale applies to all images in the respective column.

We used infrared nanoimaging to visualize the propagating polaritons in the graphene/α-MoO_3_ heterostructures (Supplementary Fig. 5) and verify the above theoretical predictions (Fig. 2a–c,g–i). In this technique, upon p-polarized infrared light illumination, the resonant gold antenna efficiently launches hybrid polaritons (Supplementary Fig. 6), originating in the nanoscale concentrated fields at the two endpoints. While scanning the sample, the real part of the scattered light electric field (Re{*E*_S_}) is recorded simultaneously with the topography, making it possible to directly map the vertical near-field components of the hybrid polariton wavefronts launched by the antenna (for more details on near-field image analysis, see Supplementary Figs. 7 and 8).

To visualize the polariton wavefronts^34,35^, we imaged antenna-launched polaritons in the heterostructure at several intermediate graphene Fermi energies in the range *E*_F_ = 0–0.7 eV (Supplementary Fig. 9). We first investigated hybrid polaritons in an undoped sample with *E*_F_ = 0 eV, which revealed a precise hyperbolic wavefront (Fig. 2a,g) for both SiO_2_ and gold substrates, consistent with previous results for a single film of α-MoO_3_ as a result of the hyperbolic dispersion contour. Next, we examined the optical response of a sample with a relatively high doping level (*E*_F_ = 0.3 eV) on a SiO_2_ substrate at the same incidence frequency. The image in Fig. 2b shows that the measured wavefronts remain hyperbolic along the *x* direction, while fringes around the antenna appear in the *y* direction, indicating that the dispersion contour has evolved into a closed shape, as shown in Fig. 1b and Supplementary Fig. 2. In contrast, the sample on a gold substrate shows a nearly flat wavefront for *E*_F_ = 0.3 eV (Fig. 2h), indicating a topological transition in the dispersion contour along the *x* direction. On increasing the doping level further to *E*_F_ = 0.7 eV, only elliptical wavefronts were observed (Fig. 2c,i) for samples on both SiO_2_ and gold substrates, denoting a rather rounded anisotropic dispersion contour. The corresponding Fourier transforms of the experimental near-field images (Fig. 2d–f,j–l) and simulated near-field distributions (Supplementary Figs. 3 and 4) further confirmed the transformation of the dispersion contour with increasing doping level of graphene. Notably, our extracted experimental polariton wave vectors *k* = 2π/*λ*_p_ (dotted symbols in Supplementary Fig. 10; *λ*_p_ is the wavelength of hybrid polaritons) match quite well the calculated dispersion diagrams in all cases.

Low levels of disorder or minor imperfections in the heterostructure should not substantially affect the control capability (Supplementary Fig. 13). In addition, the thickness of α-MoO_3_ determines the influence of the dielectric environment (here, the substrate), which should not exceed the skin depth of hybrid polaritons (Supplementary Fig. 14).

Close to *E*_F_ = 0.3 eV, where the dispersion contour is rather flat, as shown in Fig. 2h, the propagation of hybrid polaritons appears to be firmly guided along the *x* direction, yielding a highly directive and diffractionless behaviour. Furthermore, this type of polariton canalization can be found over a wide range of frequencies and different thicknesses of α-MoO_3_ (Supplementary Fig. 11) due to the inherent robustness of the topological transition. The line profiles (vertical cuts along the *y* direction) across the amplitude of the canalization mode give a full-width at half-maximum (FWHM) of around 115 ± 5 nm (~*λ*_0_/95, where *λ*_0_ is the free-space wavelength), as shown in Supplementary Fig. 12.

## Launching and manipulation of hybrid polaritons with tailored antenna

By rotating the long axis of the antenna by an angle *θ* with respect to the *y* direction, we could selectively launch and manipulate hybrid polaritons with different in-plane wave vectors. The launching contribution from our antenna can be decomposed into four parts: two endpoints acting as resonant dipoles and two parallel edges behaving as line dipoles (and assimilated to linear arrays of discrete dipoles).

**Fig. 3:**
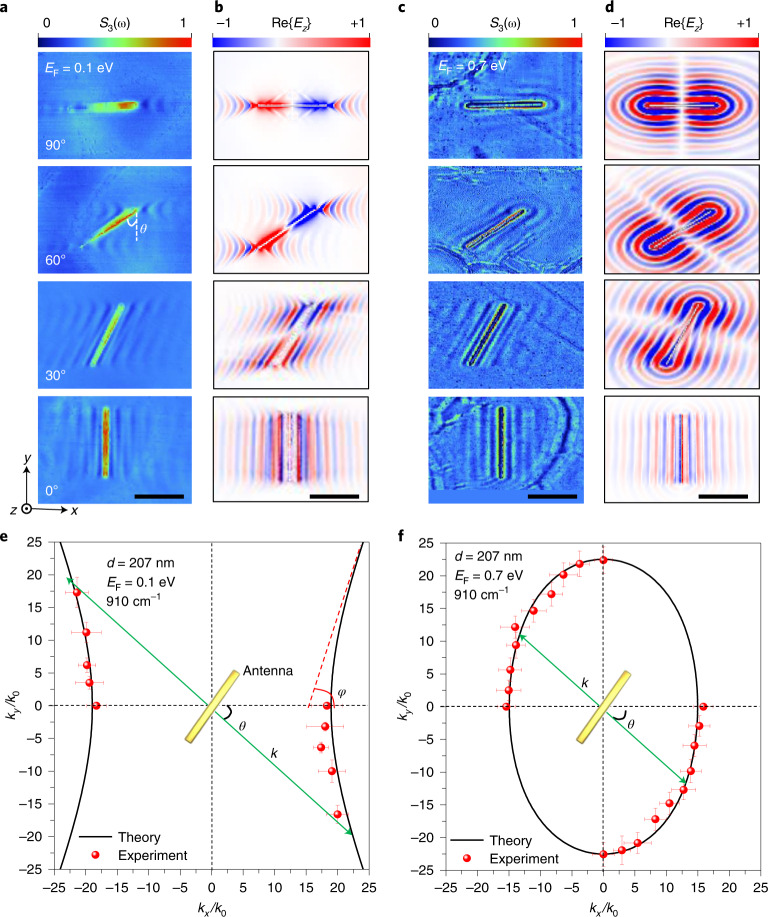
Antenna-tailored launching of hybrid polaritons. **a**,**c**, Experimentally measured near-field amplitude (*S*_3_) images of hybrid polaritons launched by gold antennas with orientation angle *θ* in the 0–90° range (Supplementary Fig. 15) for a graphene Fermi energy *E*_F_ = 0.1 eV (**a**) and *E*_F_ = 0.7 eV (**c**) at a light frequency of 910 cm^−1^. **b**,**d**, Numerically simulated near-field distributions (Re{*E*_*z*_}, evaluated 20 nm above the surface of the heterostructure) corresponding to the measured results shown in **a** and **c**, respectively. **e**,**f**, Isofrequency dispersion contours extracted from the experimental data in **a** and **c**, respectively (red symbols), compared with the calculated hyperbolic dispersion contour (black solid curves) for an opening angle *φ*. The green arrows illustrate the direction of the exciting polariton wave vector **k** perpendicular to the long axis of the gold antenna. The α-MoO_3_ thickness was 207 nm in all panels. Scale bars in **a**–**d**, 2 μm. The error bars were extracted from four sets of measurements on the in situ sample (Supplementary Fig. 15). The artefacts observed in **c** and not in **a** can be attributed to the grain boundaries of polycrystalline graphene prepared by chemical vapour deposition^43^. Source data

Figure 3a shows for a sample with a low doping level (*E*_F_ = 0.1 eV) that, at *θ* = 0°, the field pattern of the exciting hybrid polaritons exhibits vertical fringes parallel to the long axis of the antenna, dominated by the line dipole contributions generated by the edges. Note that the two endpoint dipoles are not well excited when *θ* = 0° because the polarization direction of the incident light is not aligned with the long axis of the antenna. We can extract the polariton wave vector from the fringes parallel to this long axis. As *θ* increases from 0 to 90°, the wavefronts produced by the two endpoints gradually show up and interact with those produced by the edges. In the regime with rotation angles *θ* ≤ 40°, the distance between adjacent fringes parallel to the antenna edge is reduced from 590 to 380 nm, from which we can obtain the wave vector **k** perpendicular to the antenna. The extracted wave vectors are shown by red symbols in Fig. 3e, matching quite well the calculated dispersion contour (solid curves; more details on the extraction analysis are provided in Supplementary Fig. 16). When *θ* ≥ 60° (~*φ*, the opening angle indicated in Fig. 3e), there are no fringes parallel to the edge of the antenna because polariton propagation is prohibited along that direction, judging from the dispersion contour, and the field pattern is dominated by the hyperbolic wavefronts produced by the two endpoints of the antenna. The simulated field patterns shown in Fig. 3b corroborate these experimental observations for different rotation angles.

For a sample with a high doping level (*E*_F_ = 0.7 eV, Fig. 3c), the antenna can generate polaritons propagating in all directions within the *x*–*y* plane when the polarization direction of the incident light is along the long axis of the antenna. Due to the in-plane anisotropy, the excited field patterns are therefore different for the various rotation angles explored in the range from 0 to 90°. The simulated field patterns (Fig. 3d) again agree well with our experimental observations. The polariton wave vectors can still be extracted from the measured fringes perpendicular to the antenna, shown as red symbols in Fig. 3f, which also match quite well the calculated dispersion contour (solid curves). Note that, at *E*_F_ = 0.3 eV, the field patterns of hybrid polaritons launched by antennas with different rotation angles (from 0 to 45°) all lie strictly along the *x* direction (Supplementary Fig. 17) due to the flattening of the dispersion contour, which leads to directional canalization at this graphene Fermi energy. More details on the extraction of polariton wave vectors from experiments can be found in Supplementary Figs. 16 and 18.

## Partial focusing of polaritons by substrate engineering

**Fig. 4:**
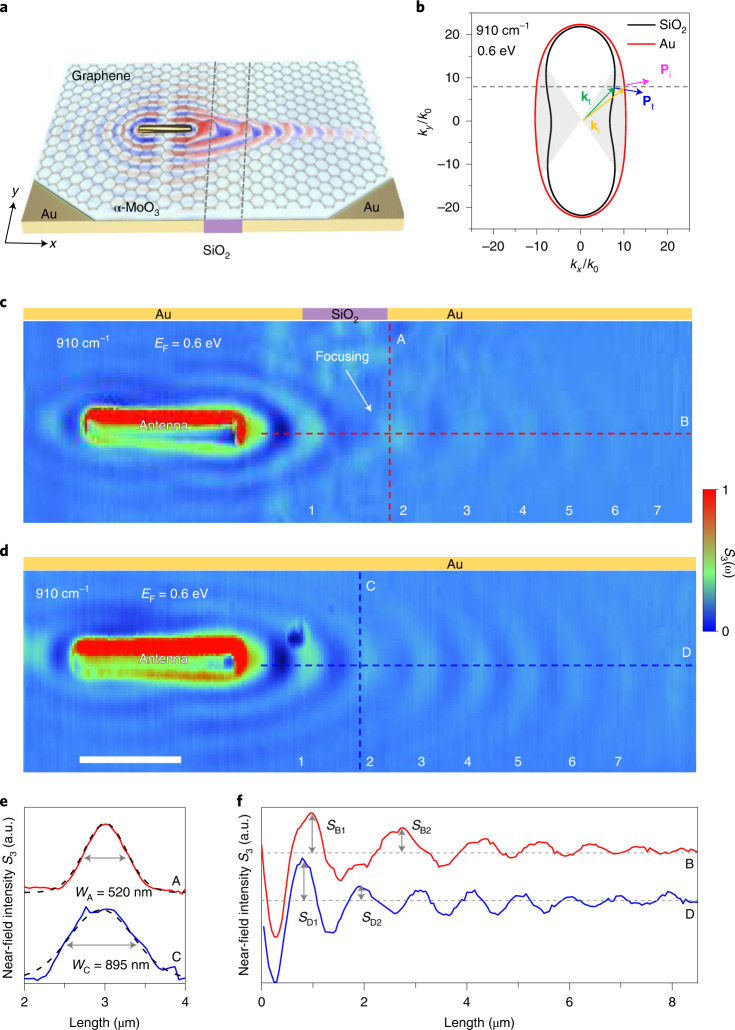
Partial focusing of hybrid polaritons by substrate engineering. **a**, Schematic of the design, where the heterostructure lies on top of a substrate composed of a Au–SiO_2_–Au in-plane sandwich structure. **b**, Isofrequency dispersion contours of hybrid polaritons for Au and SiO_2_ substrates at 910 cm^−1^ (*λ*_0_ = 10.99 μm). The shaded areas highlight the convex and concave dispersion contours in the region around the *x* axis on the gold and SiO_2_ substrates, respectively. With a wave vector inside the shaded area, negative refraction can happen at the Au–SiO_2_ interface when the polaritons on the gold substrate propagate towards that interface. The scheme for negative refraction is illustrated by further showing the incident wave vector **k**_i_ and the Poynting vector **P**_i_, together with the resulting transmitted **k**_t_ and **P**_t_. **c**, Experimentally measured near-field amplitude (*S*_3_) image of hybrid polaritons showing partial focusing in the system shown in **a**. The central SiO_2_ film was 1.5 µm wide and served as an in-plane flat lens. The antenna was located 1.0 µm away from the left Au–SiO_2_ interface. **d**, Experimentally measured hybrid polaritons on a Au substrate, as a control to **c**. Scale bar indicates 1.5 μm and also applies to **c**. **e**, Near-field profiles for the sections marked by the red (A) and blue (C) vertical dashed lines in **c** and **d**, respectively. The black dashed curves are Gaussian fittings. *W*_A_ and *W*_C_ indicate the full width at half maximum (FWHM) of profiles A and C, respectively. **f**, Near-field profiles of the sections marked by red (B) and blue (D) horizontal dashed lines in **c** and **d**, respectively. *S*_B1_, *S*_B2_, *S*_D1_ and *S*_D2_ represent the electric-field intensity at each fringe. The graphene was doped to *E*_F_ = 0.6 eV and the α-MoO_3_ thickness was 320 nm.

As the dispersion contour of the hybrid polaritons can be modified substantially by controlling the dielectric environment, we engineered the substrate for the heterostructure to manipulate the in-plane propagation of hybrid polaritons. The design is illustrated in Fig. 4a, where the heterostructure lies on top of a substrate composed of a Au–SiO_2_–Au in-plane sandwich structure. This substrate was used to locally engineer the isofrequency dispersion contour (Fig. 4b). The central SiO_2_ film, with a width of 1.5 μm, serves as an in-plane flat lens to partially focus the incident polaritons (with a wave vector **k**_i_ and a Poynting vector **P**_i_ along the normal of the contour in Fig. 4b) generated by an antenna on top of the left gold substrate. When the hybrid polaritons cross the boundary between the gold and SiO_2_ substrates, due to the change in the detailed shape of the dispersion contour, negative refraction can occur at the boundary, with the *y* component of the wave vector being conserved, whereas the sign of the *y* component of the transmitted Poynting vector **P**_t_ is opposite to that of the incident **P**_i_, as illustrated in Fig. 4b. This leads to a partial focusing of the polaritons (Fig. 4c). Supplementary Fig. 19 shows the evolution of the negative refraction of hybrid polaritons at different Fermi energies of graphene.

The measured antenna-launched polariton near-field distributions for the heterostructure on gold and SiO_2_ substrates are shown in Fig. 4d and Supplementary Figs. 20 and 21, respectively. On the gold substrate, only elliptical wavefronts are observed around the antenna, denoting a convex dispersion contour in the region around the *x* axis. In contrast, on the SiO_2_ substrate (Supplementary Fig. 21), wavefronts are hyperbolic along the *x* direction and elliptic along the *y* direction around the antenna, indicating a closed concave shape of the dispersion contour near the *x* axis. These measurements are consistent with our previous experimental results shown in Figs. 2 and 3, and also, they match quite well the isofrequency contours shown in Fig. 4b.

Furthermore, we launched hybrid polaritons towards the Au–SiO_2_–Au in-plane sandwich substrate from the left gold part by light coupling to an antenna prepared in that region. The resulting near-field distribution is shown in Fig. 4c (for more details, see Supplementary Figs. 20 and 21). When polaritons having elliptical wavefronts on the left gold substrate cross the boundary between the gold and SiO_2_ substrates, the Poynting vector of the polaritons refracts on the same side of the boundary-normal direction, therefore producing what is known as negative refraction due to the change in the detailed shape of the dispersion contour, which ultimately leads to partial focusing of the incident polaritons. Indeed, Fig. 4c shows the formation of a focal spot close to the right Au–SiO_2_ interface. The numerically simulated *z* out-of-plane component of the electric field distributions (Re{*E*_*z*_}) shown in Supplementary Fig. 22 further corroborate the experimental findings. The red curve in Fig. 4e shows the spatial distribution of the electric field amplitude at the focal spot, demonstrating a high wavelength compression towards a FWHM of 520 nm along the *y* direction. This focal spot size is less than 1/21 of the corresponding illumination wavelength, thus emphasizing a deep subwavelength focusing effect (for more details, see Supplementary Figs. 23 and 24).

To estimate the intensity enhancement of the observed partial focusing, we extracted the spatial distribution of the electric field from the propagation profile (Fig. 4f). The intensity enhancement *ξ* is given by the square of the ratio of the electric field at the focal spot to that without focusing, $\xi = \left( {\frac{{S_{{\mathrm{B}}2}/S_{{\mathrm{B}}1}}}{{S_{{\mathrm{D}}2}/S_{{\mathrm{D}}1}}}} \right)^2 = 4.5$. *S*_B1_, *S*_B2_, *S*_D1_, and *S*_D2_ represent the near-field intensity at each fringe. Note that the focusing effect can be further enhanced by improving the flatness of the interface, as its structural inhomogeneity inevitably introduces undesired reflection, scattering and radiative losses of the incident polaritons (for more details, see Supplementary Figs. 25–27).

## Conclusions

We have experimentally demonstrated that the topology of the isofrequency dispersion contours for the hybrid polaritons supported in a heterostructure composed of a graphene sheet on top of an α-MoO_3_ layer can be substantially modified by chemically changing the doping level of graphene, with the contour topology being transformed from open to closed shapes over a broad frequency range. A flat dispersion contour appears at the topological transition, which supports a highly directive and diffractionless polariton propagation, resulting in a tunable canalization mode controlled by the doping level of graphene. We anticipate that electrical gating could be used to control the doping level in future studies^36,37^. Furthermore, through the appropriate choice of substrate for the heterostructure, we were also able to engineer the dispersion contour to exhibit even flatter profiles (for example, by using a gold substrate). This property has allowed us to design a deep-subwavelength device for in-plane focusing of hybrid polaritons, where negative refractive occurs at the boundary between two different substrates. Our study opens promising possibilities to tune topological polaritonic transitions in low-dimensional materials^38–40^ with potential applications in optical imaging, sensing and the control of spontaneous emission at the nanoscale^18^.

*Note added in proof*: While preparing this manuscript, two related theoretical and experimental studies on the tuning of highly anisotropic phonon polaritons in graphene and α-MoO_3_ vdW structures were reported^41,42^.

## Methods

### Nanofabrication of the devices

High-quality α-MoO_3_ flakes were mechanically exfoliated from bulk crystals synthesized by the chemical vapour deposition (CVD) method^19^ and then transferred onto either commercial 300 nm SiO_2_/500 μm Si wafers (SVM) or gold substrates using a deterministic dry transfer process with a polydimethylsiloxane (PDMS) stamp. CVD-grown monolayer graphene on copper foil was transferred onto the α-MoO_3_ samples using the poly(methyl methacrylate) (PMMA)-assisted method following our previous report^44^.

The launching efficiency of the resonant antenna is mainly determined by its geometry, together with a trade-off between the optimum size and illumination frequency^45,46^. We designed the gold antenna with a length of 3 μm and a thickness of 50 nm, which provided a high launching efficiency over the spectral range from 890 to 950 cm^−1^ within the α-MoO_3_ reststrahlen band. Alternatively, a thicker antenna with a stronger *z* component of the electric field could be used to launch the polaritons more efficiently in future studies. Note that narrow antennas (50-nm width) were used to prevent their shapes from affecting the polariton wavefronts, especially when their propagation is canalized (such as in Figs. 2 and 3), while wider antennas (250-nm width) were used to obtain a higher launching efficiency and observe polaritons propagating across the SiO_2_–Au interface in our experiments (for example, Fig. 4).

Gold antenna arrays were patterned on selected α-MoO_3_ flakes using 100 kV electron-beam lithography (Vistec 5000+ES) on an approximately 350 nm PMMA950K lithography resist. Electron-beam evaporation was subsequently used to deposit 5 nm Ti and 50 nm Au in a vacuum chamber at a pressure of <5 × 10^−6^ torr to fabricate the Au antennas. Electron-beam evaporation was also used to deposit a 60-nm-thick gold film onto a low-doped Si substrate. To remove any residual organic material, samples were immersed in a hot acetone bath at 80 °C for 25 min and then subjected to gentle rinsing with isopropyl alcohol (IPA) for 3 min, followed by drying with nitrogen gas and thermal baking (for more details on the fabrication and characterization of the Au–SiO_2_–Au in-plane sandwich structure, see Supplementary Figs. 26 and 27).

The samples were annealed in a vacuum to remove most of the dopants from the wet transfer process and then transferred to a chamber filled with NO_2_ gas to achieve different doping levels by surface adsorption of gas molecules^47^. The graphene Fermi energy could be controlled by varying the gas concentration and doping time, achieving values as high as ~0.7 eV (Supplementary Fig. 9). This gas-doping method provides excellent uniformity, reversibility and stability. Indeed, Raman mapping of a gas-doped graphene sample demonstrated the high uniformity of the method (Supplementary Fig. 9). As the deposition of NO_2_ gas molecules on the graphene surface occurs by physical adsorption, the topological transition of hybrid polaritons in graphene/α-MoO_3_ heterostructures can be reversed by controlling the gas doping. For example, after gas doping, the Fermi energy of graphene could be lowered from 0.7 to 0 eV by vacuum annealing at 150 °C for 2 h. The sample could subsequently be re-doped to reach another on-demand Fermi energy (Supplementary Fig. 28). It should be noted that the graphene Fermi energy only decreases from 0.7 to 0.6 eV after being left for 2 weeks under ambient conditions, which demonstrates the high stability of the doping effect (Supplementary Fig. 29). Note that chemical doping has been demonstrated to be an effective way to tune the characteristics of polaritons, such as their strength and in-plane wavelength^48–51^.

### Scanning near-field optical microscopy measurements

A scattering scanning near-field optical microscope (Neaspec) equipped with a wavelength-tunable quantum cascade laser (890–2,000 cm^−1^) was used to image optical near fields. The atomic force microscopy (AFM) tip of the microscope was coated with gold, resulting in an apex radius of ~25 nm (NanoWorld), and the tip-tapping frequency and amplitudes were set to ~270 kHz and ~30–50 nm, respectively. The laser beam was directed towards the AFM tip, with lateral spot sizes of ~25 μm under the tip, which were sufficient to cover the antennas as well as a large area of the graphene/α-MoO_3_ samples. Third-order harmonic demodulation was applied to the near-field amplitude images to strongly suppress background noise.

In our experiments, the p-polarized plane-wave illumination (electric field **E**_inc_) impinged at an angle of 60° relative to the tip axis^52^. To avert any effects caused by the light polarization direction relative to the crystallographic orientation of α-MoO_3_, which is optically anisotropic, the in-plane projection of the polarization vector coincided with the *x* direction ([100] crystal axis) of α-MoO_3_ (Supplementary Fig. 6). Supplementary Fig. 30 shows the method used to extract antenna-launched hybrid polaritons from the complex background signals observed when the polaritons propagate across a Au–SiO_2_–Au in-plane structure to realize partial focusing.

### Calculation of polariton dispersion and isofrequency dispersion contours (IFCs) of hybrid plasmon–phonon polaritons

The transfer matrix method was adopted to calculate the dispersion and IFCs of hybrid plasmon–phonon polaritons in graphene/α-MoO_3_ heterostructures. Our theoretical model was based on a three-layer structure: layer 1 (*z* > 0, air) is a cover layer, layer 2 (0 > *z* > –*d*_h_, graphene/α-MoO_3_) is an intermediate layer and layer 3 (*z* < –*d*_h_, SiO_2_ or Au) is a substrate where *z* is the value of the vertical axis and *d*_h_ is the thickness of α-MoO_3_ (Supplementary Fig. 31). Each layer was regarded as a homogeneous material represented by the corresponding dielectric tensor. The air and substrate layers were modelled by isotropic tensors diag{*ε*_a,s_} (ref. ^53^). The α-MoO_3_ film was modelled by an anisotropic diagonal tensor diag{*ε*_*x*_, *ε*_*y*_, *ε*_*z*_}, where *ε*_*x*_, *ε*_*y*_ and *ε*_*z*_ are the permittivity components along the *x*, *y* and *z* axes, respectively. Also, monolayer graphene was located on top of α-MoO_3_ at *z* = 0 and described as a zero-thickness current layer characterized by a frequency-dependent surface conductivity taken from the local random-phase approximation model^54,55^:

1 $$\begin{array}{rcl} {\sigma \left( \omega \right)}& = &{\frac{{i{{\rm{e}}^2}{k_{\rm{B}}}T}}{{{{\uppi}}{\hbar ^2}\left( {\omega + \frac i \tau} \right)}}\left[ {\frac{{{E_{\rm{F}}}}}{{{k_{\rm{B}}}T}} + 2\ln \left( {{{\rm{e}}^{ - \frac{{{E_{\rm{F}}}}}{{{k_{\rm{B}}}T}}}} + 1} \right)} \right]}\\{}&{}&{ + i\frac{{{{\rm{e}}^2}}}{{4{{\uppi}}\hbar }}\ln \left[ {\frac{{2\left| {{E_{\rm{F}}}} \right| - \hbar \left( {\omega + \frac i \tau} \right)}}{{2\left| {{E_{\rm{F}}}} \right| + \hbar \left( {\omega + \frac i \tau} \right)}}} \right]}\end{array}$$

which depends on the Fermi energy *E*_F_, the inelastic relaxation time *τ* and the temperature T; the relaxation time is expressed in terms of the graphene Fermi velocity *v*_F_ = *c*/300 and the carrier mobility *μ*, with $\tau = \mu E_{\mathrm{F}}/ev_{\mathrm{F}}^2$; *e* is the elementary charge; *k*_B_ is the Boltzmann constant; *ℏ* is the reduced Planck constant; and *ω* is the illumination frequency.

Given the strong field confinement produced by the structure under consideration, we only needed to consider transverse magnetic (TM) modes, because transverse electric (TE) components contribute negligibly. The corresponding p-polarization Fresnel reflection coefficient *r*_p_ of the three-layer system admits the analytical expression

2 $$\begin{array}{*{20}{c}} {r_{\mathrm{p}} = \frac{{r_{12} + r_{23}\left( {1 - r_{12} - r_{21}} \right){\mathrm{e}}^{i2k_z^{\left( 2 \right)}d_{\mathrm{h}}}}}{{1 + r_{12}r_{23}{\mathrm{e}}^{i2k_z^{\left( 2 \right)}d_{\mathrm{h}}}}},} \end{array}$$

3 $$\begin{array}{*{20}{c}} {r_{12} = \frac{{{{Q}}_1 - {{Q}}_2 + SQ_1Q_2}}{{{{Q}}_1 + Q_2 + SQ_1Q_2}},} \end{array}$$

4 $$\begin{array}{*{20}{c}} {r_{21} = \frac{{{{Q}}_2 - {{Q}}_1 + SQ_1Q_2}}{{{{Q}}_2 + Q_1 + SQ_1Q_2}},} \end{array}$$

5 $$\begin{array}{*{20}{c}} {r_{23} = \frac{{Q_2 - Q_3}}{{Q_2 + Q_3}},} \end{array}$$

6 $$\begin{array}{*{20}{c}}where {Q_j = \frac{{k_z^{\left( j \right)}}}{{{\it{\epsilon }}_t^{(j)}}},} \end{array}$$

7 $$\begin{array}{*{20}{c}} {S = \frac{{\sigma Z_0}}{\omega }.} \end{array}$$

Here, *r*_*jk*_ denotes the reflection coefficient of the *j*–*k* interface for illumination from medium *j*, with *j*,*k* = 1–3; ${\it{\epsilon }}_t^{(j)}$ is the tangential component of the in-plane dielectric function of layer *j* for a propagation wave vector *k*_p_(*θ*) (where *θ* is the angle relative to the *x* axis), which can be expressed as ${\it{\epsilon }}_t^{(j)} = {\it{\epsilon }}_x^{(j)}\mathop {{\cos }}\nolimits^2 \theta + {\it{\epsilon }}_y^{(j)}\mathop {{\sin }}\nolimits^2 \theta$ (where ${\it{\epsilon }}_x^{(j)}$ and ${\it{\epsilon }}_y^{(j)}$ are the diagonal dielectric tensor components of layer *j* along the *x* and *y* axes, respectively); $k_z^{\left( j \right)} = \sqrt {\varepsilon _t^{\left( j \right)}\frac{{\omega ^2}}{{c^2}} - \frac{{\varepsilon _t^{\left( j \right)}}}{{\varepsilon _z^{\left( j \right)}}}q^2}$ is the out-of-plane wave vector, with ${\it{\epsilon }}_z^{(j)}$ being the dielectric function of layer *j* along the *z* axis; and *Z*_0_ is the vacuum impedance.

We find the polariton dispersion relation *q*(*ω*,*θ*) when the denominator of equation (2) is zero:

8 $$\begin{array}{*{20}{c}} {1 + r_{12}r_{23}{\mathrm{e}}^{i2k_z^{\left( 2 \right)}d_{\mathrm{h}}} = 0.} \end{array}$$

For simplicity, we considered a system with small dissipation, so that the maxima of Im{*r*_p_} (see colour plots in Supplementary Figure 10) approximately solve the condition given by equation (8), and therefore produce the sought-after dispersion relation *q*(*ω*,*θ*) (see additional discussion in Supplementary Note 1).

### Electromagnetic simulations

The electromagnetic fields around the antennas were calculated by a finite-elements method using the COMSOL package. In our experiments, both tip and antenna launching were investigated. For the former, the sharp metallic tip was illuminated by an incident laser beam. The tip acted as a vertical optical antenna, converting the incident light into a strongly confined near field below the tip apex, which can be regarded as a vertically oriented point dipole located at the tip apex. This localized near field provided the necessary momentum to excite polaritons. Consequently, we modelled the tip as a vertical *z*-oriented oscillating dipole in our simulations (Fig. 1c,f), a procedure that has been widely used for tip-launched polaritons in vdW materials^56^. For the antenna launching, the gold antenna can provide strong near fields of opposite polarity at the two endpoints, thus delivering high-momentum near-field components that match the wave vector of the polaritons and excite propagating modes in the graphene/α-MoO_3_ heterostructure^45,46^. Our simulations of polariton excitation by means of antennas, such as in Fig. 3b,d, incorporated the same geometrical design as in the experimental structures.

We also used a dipole polarized along the *z* direction to launch polaritons, and the distance between the dipole and the uppermost surface of the sample was set to 100 nm. We obtained the distribution of the real part of the out-of-plane electric field (Re{*E*_*z*_}) over a plane 20 nm above the surface of graphene. The boundary conditions were set to perfectly matching layers. Graphene was modelled as a transition interface with a conductivity described by the local random-phase approximation model (see above)^55,57^. We assumed a graphene carrier mobility of 2,000 cm^2^ V^–1^ s^–1^. Supplementary Fig. 1c,d shows the permittivity of SiO_2_ and Au, respectively, at the mid-infrared wavelengths used.

## Online content

Methods, additional references, Nature Research reporting summaries, source data, extended data, supplementary information, acknowledgements, peer review information; details of author contributions and competing interests; and statements of data and code availability are available at 10.1038/s41565-022-01185-2.

## Supplementary information

Supplementary Information Supplementary Figs. 1–31.

## Data Availability

The data that support the findings of this study are available within the paper and the Supplementary Information. Other relevant data are available from the corresponding authors upon reasonable request. Source data are provided with this paper.

## Source data

Source Data Fig. 3 Statistical source data.

## Code availability

The code that support the findings of this study are available from the corresponding authors upon reasonable request.
