# Supplementary material for: Doping-driven topological polaritons in graphene/α-MoO3 heterostructures
Source: Nat Nanotechnol. 2022 Aug 18;17(9):940–6. doi: 10.1038/s41565-022-01185-2 (PMC9477736; doi:10.1038/s41565-022-01185-2)
Supplement: Supplementary file 1 — Supplementary Figs. 1–31. [file 41565_2022_1185_MOESM1_ESM.pdf]

nature portfolio

https://doi.org/10.1038/s41565-022-01185-2

Supplementary information

Doping-driven topological polaritons in graphene/α-MoO_3 heterostructures

In the format provided by the authors and unedited

Supplementary Information for

Doping-driven topological polaritons in graphene/α-MoO_3 heterostructures

Hai Hu,[1, 2†*] Na Chen,[1,2†] Hanchao Teng,[1, 2†] Renwen Yu,[3, 4*] Yunpeng Qu, [1, 2] Jianzhe Sun,[7] Mengfei Xue,[8] Debo Hu,[1, 2] Bin Wu,[7] Chi Li,[1, 2] Jianing Chen,[8] Mengkun Liu,[9] Zhipei Sun,[10] Yunqi Liu,[7] Peining Li,[6] Shanhui Fan,[4] F. Javier García de Abajo,[3, 5*] Qing Dai[1, 2*]

1 CAS Key Laboratory of Nanophotonic Materials and Devices, CAS Key Laboratory of Standardization and Measurement for Nanotechnology, CAS Center for Excellence in Nanoscience, National Center for Nanoscience and Technology, Beijing 100190, P. R. China.
2 University of Chinese Academy of Sciences, Beijing 100049, P. R. China.
3 ICFO-Institut de Ciencies Fotoniques, The Barcelona Institute of Science and Technology, 08860 Castelldefels (Barcelona), Spain.
4 Department of Electrical Engineering, Ginzton Laboratory, Stanford University, Stanford, California 94305, United States.
5 ICREA-Institució Catalana de Recerca i Estudis Avançats, Passeig Lluís Companys 23, 08010 Barcelona, Spain.
6 Wuhan National Laboratory for Optoelectronics and School of Optical and Electronic Information, Huazhong University of Science and Technology, Wuhan, P. R. China.
7 Beijing National Laboratory for Molecular Sciences, Key Laboratory of Organic Solids, Institute of Chemistry, Beijing 100190, P. R. China.
8 The Institute of Physics, Chinese Academy of Sciences, P.O. Box 603, Beijing, China.
9 Department of Physics and Astronomy, Stony Brook University, Stony Brook, New York 11794, USA.
10 Department of Electronics and Nanoengineering Aalto University Tietotie 3, FI-02150 Espoo, Finland.

*e-mail: daiq@nanoctr.cn, javier.garciadeabajo@nanophotonics.es, renwen.yu@icloud.com, huh@nanoctr.cn
† These authors contributed equally

This PDF file includes:

Supplementary Figure 1. Optical parameters of the materials in this work.
Supplementary Figure 2. Theoretically calculated isofrequency contours of hybrid polaritons at different illumination frequencies and graphene Fermi energies.
Supplementary Figure 3. Numerically simulated field distribution of hybrid polaritons on a 300 nm SiO_2 substrate with different graphene Fermi energies.
Supplementary Figure 4. Numerically simulated field distribution of hybrid polaritons on a 60 nm gold substrate with different graphene Fermi energies.
Supplementary Figure 5. Optical image of graphene/α-MoO_3 heterostructures.
Supplementary Figure 6. Schematics of near-field nanoimaging of polaritons.
Supplementary Figure 7. Real-space infrared nanoimages reveal hybrid polaritons on the 300 nm SiO_2 substrate with different graphene Fermi energies and various illumination frequencies.
Supplementary Figure 8. Real-space infrared nanoimages reveal hybrid polaritons on the 60 nm Au substrate with different graphene Fermi energies and various illumination frequencies.
Supplementary Figure 9. Gas doping and Raman measurements of graphene samples.
Supplementary Figure 10. Dispersions of hybrid polaritons.
Supplementary Figure 11. Polariton canalizations near the topological transition at different illumination frequencies.
Supplementary Figure 12. The full width at half maximum (FWHM) of polariton canalizations.
Supplementary Figure 13. Real-space infrared nanoimages reveal hybrid polaritons in a sample with disorder.
Supplementary Figure 14. Numerically simulated field distribution of the *z* component of hybrid polaritons at different Fermi energies of graphene along with *x* (a) and *y* in-plane directions (b).
Note 1. Mode analysis of hybrid polaritons from the coupling between graphene plasmons and phonon polaritons in α-MoO_3.
Supplementary Figure 15. Near-field imaging of antenna-launched polaritons in a sample with four sets of repeated antenna arrays.
Supplementary Figure 16. Extraction analysis of antenna-tailored launching of hybrid polaritons.
Supplementary Figure 17. Numerically simulated field distribution of hybrid polaritons launched by gold antennas with different antenna angles.
Supplementary Figure 18. Reconstruction of isofrequency contours by launching hybrid polaritons in samples with defects.
Supplementary Figure 19. Numerically simulated partial focusing of hybrid polaritons based on SiO_2 and air lenses with different Fermi energies of graphene from E_F = 0.1 to 0.5 eV.
Supplementary Figure 20. Near-field characterization of graphene/α-MoO_3 on the Au-SiO_2-Au in-plane sandwich structure before doping.
Supplementary Figure 21. Partial focusing of the sample in Supplementary Figure 20a at E_F=0.6 eV.
Supplementary Figure 22. Numerically simulated field distributions of partial focusing.
Supplementary Figure 23. Partial focusing of a sample at E_F=0.6 eV.
Supplementary Figure 24. Partial focusing of sample in Supplementary Figure 23 at E_F=0.5 eV.
Supplementary Figure 25. Numerically simulated partial focusing of hybrid polaritons with different heights of the air gap from 0 to 60 nm.
Supplementary Figure 26. Fabrication process of the in-plane Au-SiO_2-Au sandwich structure.
Supplementary Figure 27. Characterization of the Au-SiO_2-Au in-plane sandwich structure by SEM and AFM.
Supplementary Figure 28. Reversibility of gas doping for topological polaritons.
Supplementary Figure 29. Stability of gas doping for topological polaritons.
Supplementary Figure 30. Method to extract antenna-launched hybrid polaritons.
Supplementary Figure 31. Illustration of the geometry considered for the theoretical model.

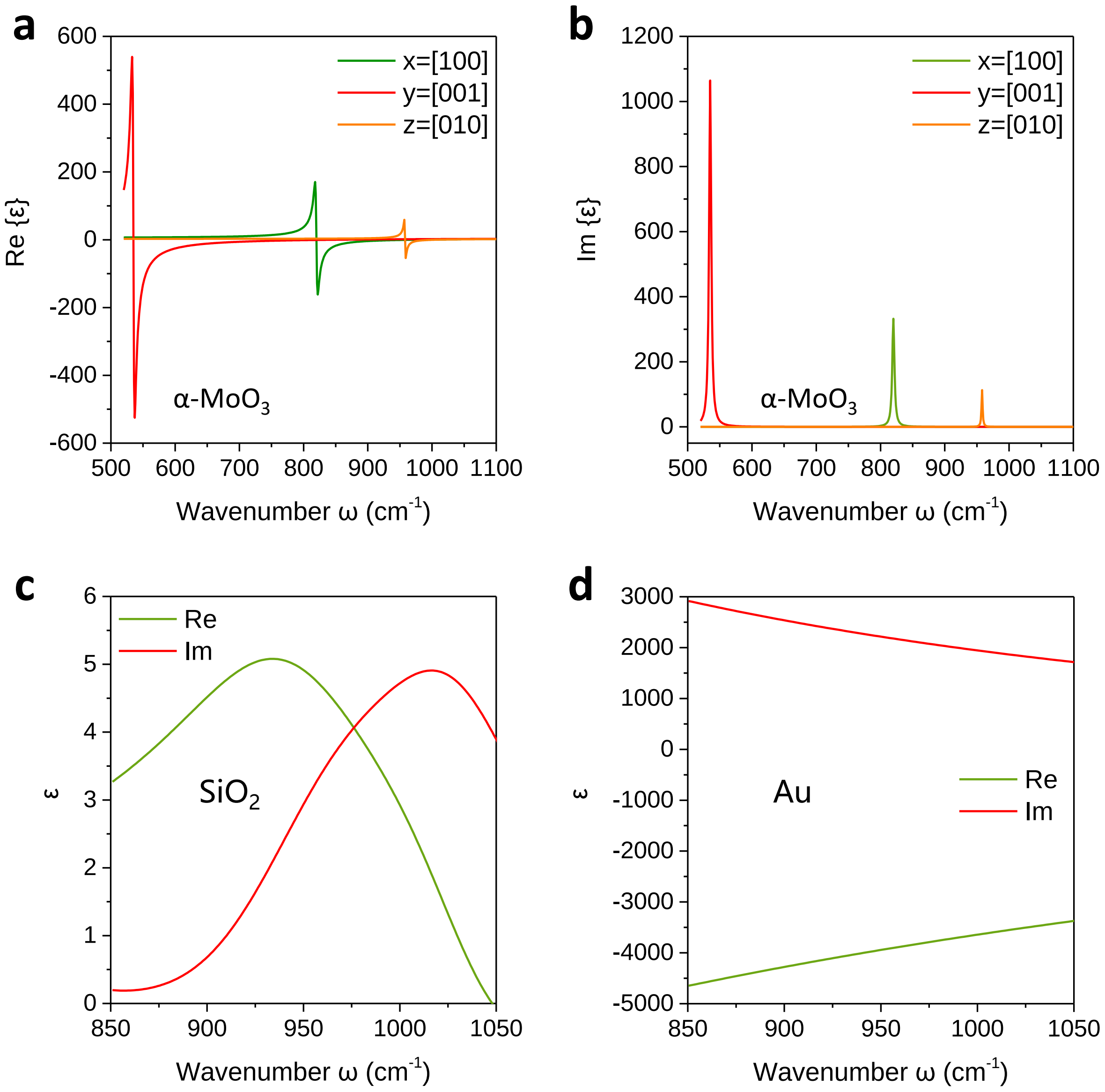

Supplementary Figure 1. Optical parameters of the materials in this work. (a, b) Permittivity of α-MoO_3 along different principal directions. The permittivity is obtained by fitting the obtained data with Lorentzian models[1]. **(c, d)** Permittivity of SiO_2 and gold[2, 3].

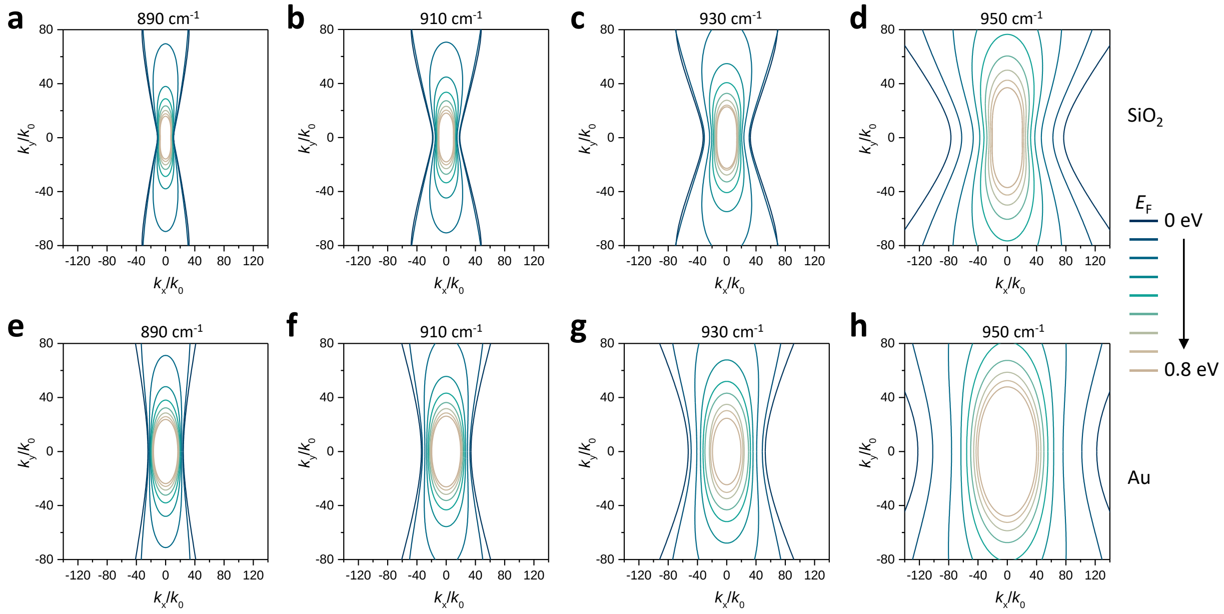

Supplementary Figure 2. Theoretically calculated isofrequency contours of hybrid polaritons at different illumination frequencies and graphene Fermi energies. (a-d) 300 nm SiO_2 substrate. (**e-h**) 60 nm gold substrate.

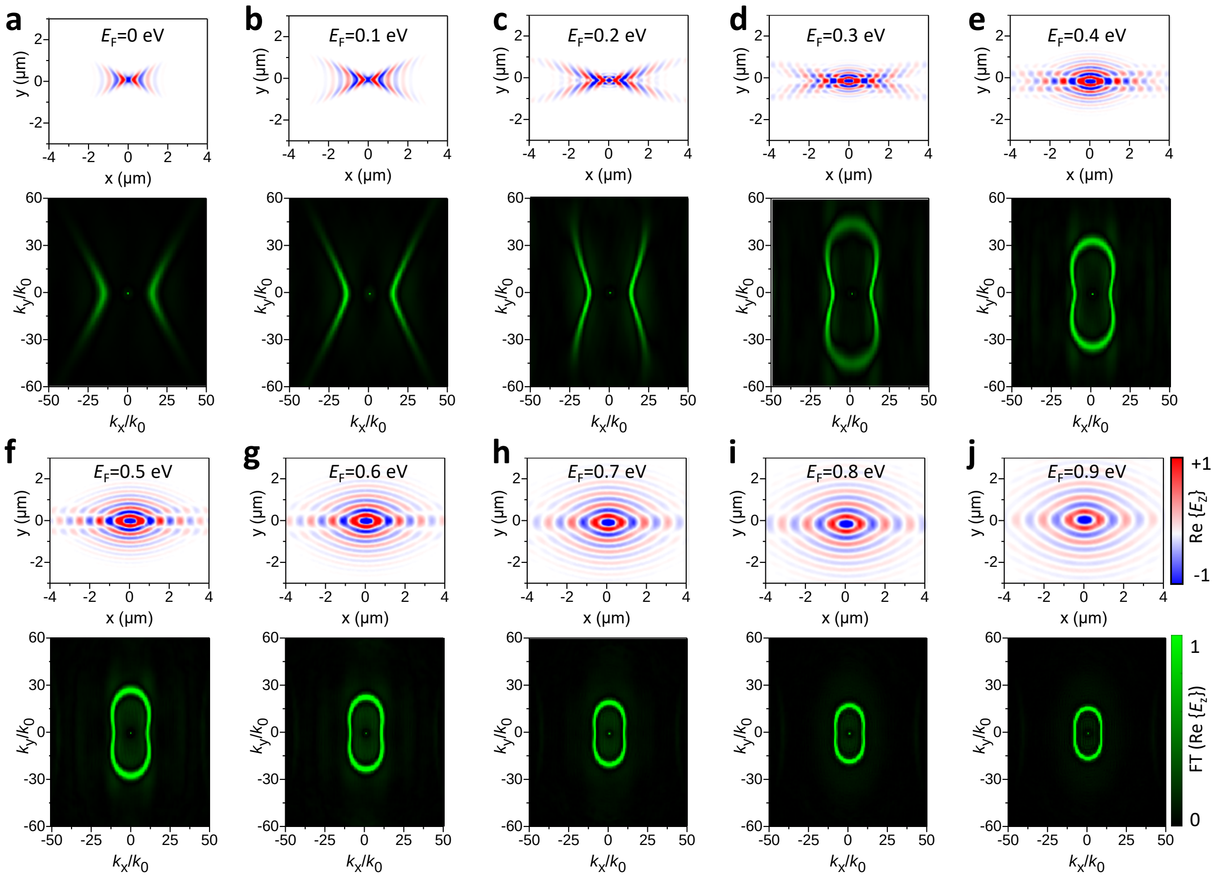

Supplementary Figure 3. Numerically simulated field distribution of hybrid polaritons on a 300 nm SiO_2 substrate with different graphene Fermi energies. A dipole is placed 100 nm above the graphene to launch the polaritons. The electric-field distribution is obtained over a plane situated 20 nm above the graphene. The graphene Fermi energy is varied from 0 to 0.9 eV (see labels), the thickness of α-MoO_3 is 150 nm, and the incident light wavelength is fixed at λ_0 = 10.99 μm (910 cm^{-1}) in all simulations.

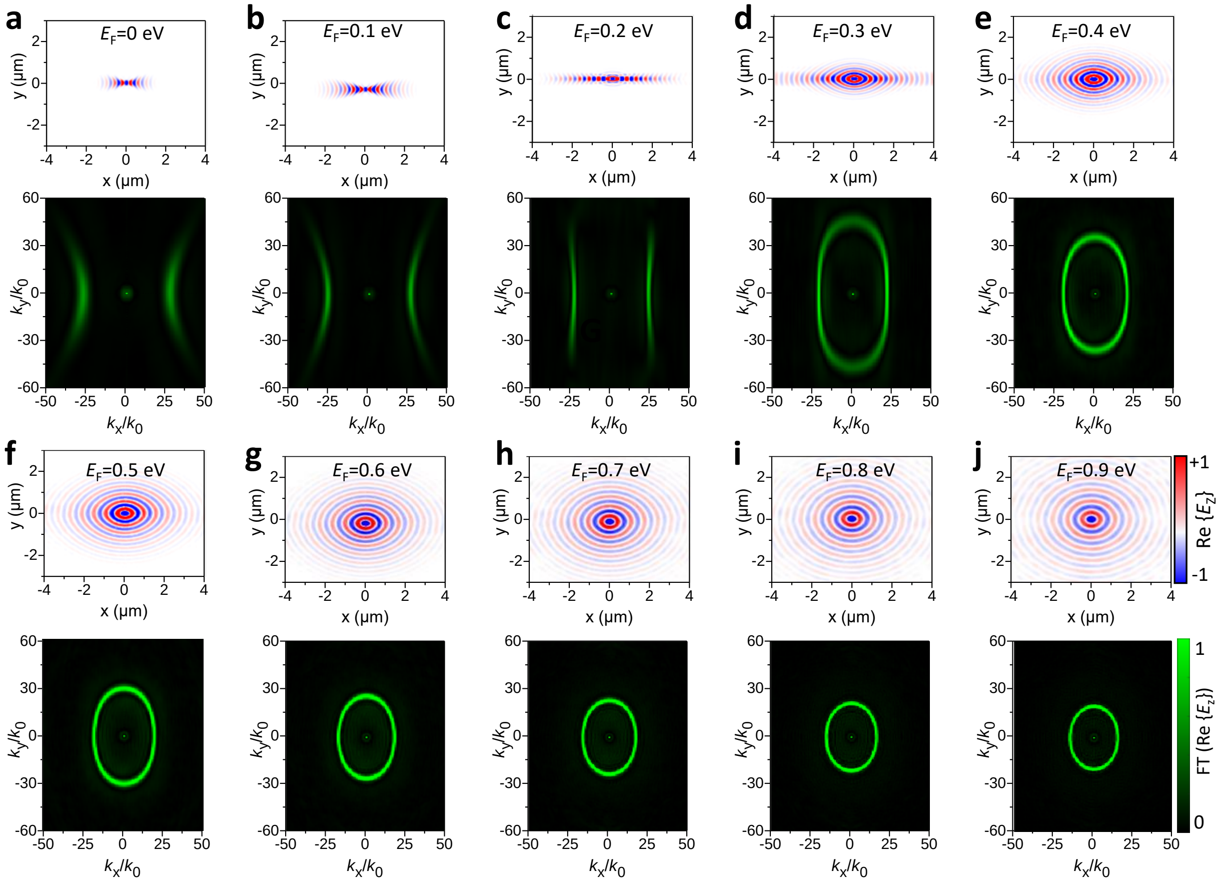

Supplementary Figure 4. Numerically simulated field distribution of hybrid polaritons on a 60 nm gold substrate with different graphene Fermi energies. A dipole is placed 100 nm above the graphene to launch the polaritons. The electric-field distribution is obtained over a plane situated 20 nm above the graphene. The graphene Fermi energy is varied from 0 to 0.9 eV (see labels), the thickness of α-MoO_3 is 150 nm, and the incident light wavelength is fixed at $\lambda_0 = 10.99$ μm (910 cm^{-1}) in all simulations.

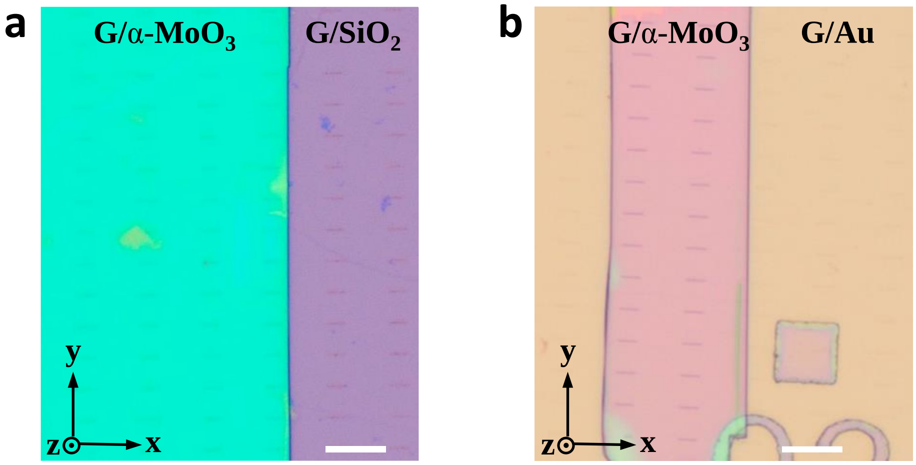

Supplementary Figure 5. Optical image of graphene/α-MoO_3 heterostructures. The samples are supported by either a 300 nm SiO_2 substrate (**a**, α-MoO_3 thickness d=140 nm) or a 60 nm gold substrate (**b**, α-MoO_3 thickness d=140 nm) and feature gold antenna arrays. The scale bar indicates 10 μm.

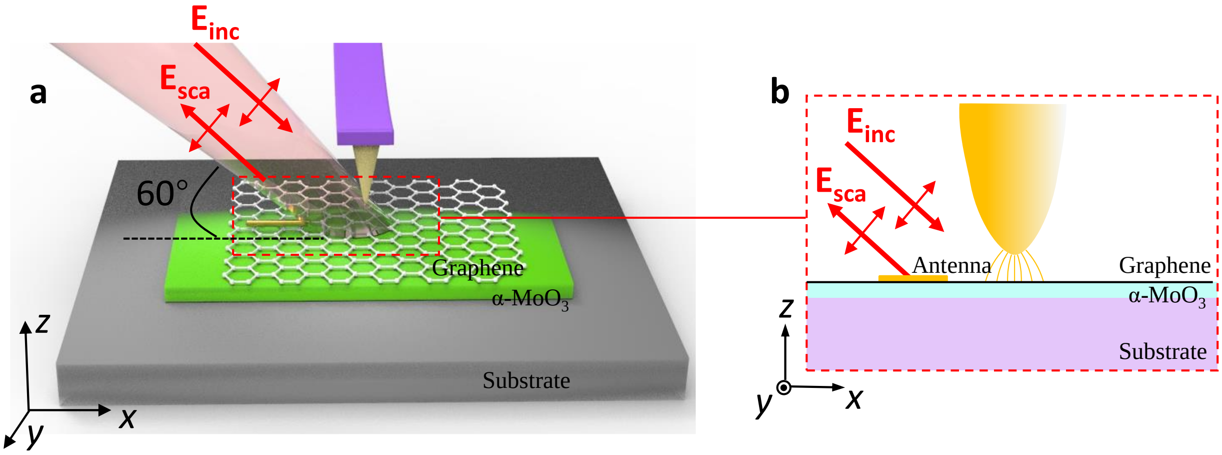

Supplementary Figure 6. Schematics of near-field nanoimaging of polaritons. Illustration of the s-SNOM setup (**a**) and schematic of incident laser light (**b**) used in this work. The p-polarized plane-wave illumination (electric field $\mathbf{E}_{inc}$) impinges at an angle of 60° relative to the substrate plane. A gold antenna (yellow) confines infrared light that allows launching polaritons in the sample, which are subsequently probed by a metalized AFM tip and then scattered into free space for collection by a distant detector.

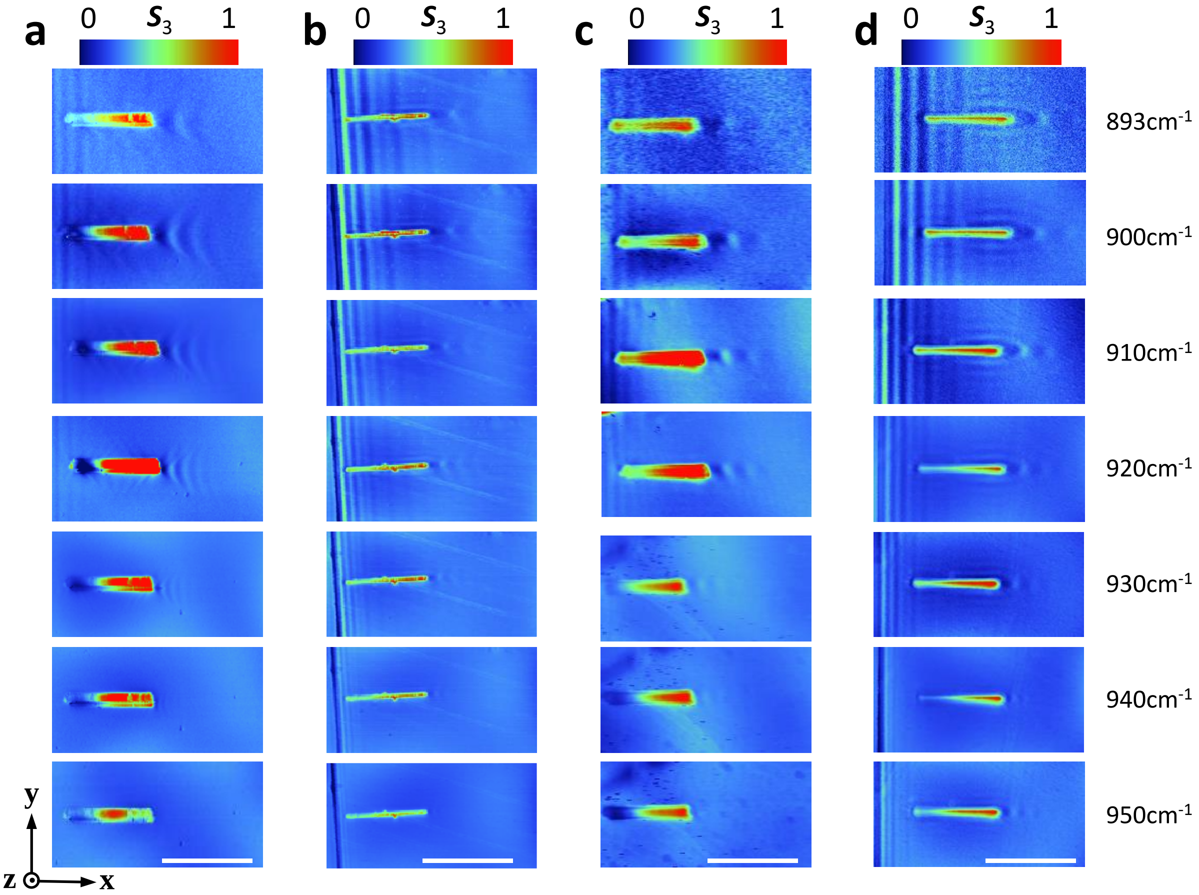

Supplementary Figure 7. Real-space infrared nanoimages reveal hybrid polaritons on the 300 nm SiO_2 substrate with different graphene Fermi energies and various illumination frequencies. (**a-d**) Antenna-launched hybrid polaritons measured in a heterostructure sample consisting of a 140-nm-thick α-MoO_3 film and monolayer graphene. The graphene Fermi energy is set at E_F=0 eV (**a**), E_F=0.3 eV (**b**), E_F=0.4 eV (**c**), and E_F=0.7 eV (**d**), respectively. The incident light wavelength is tuned from λ_0 = 11.20 μm (893 cm^{-1}) to 10.53 μm (950 cm^{-1}) in the experiments. The scale bars indicate 3 μm.

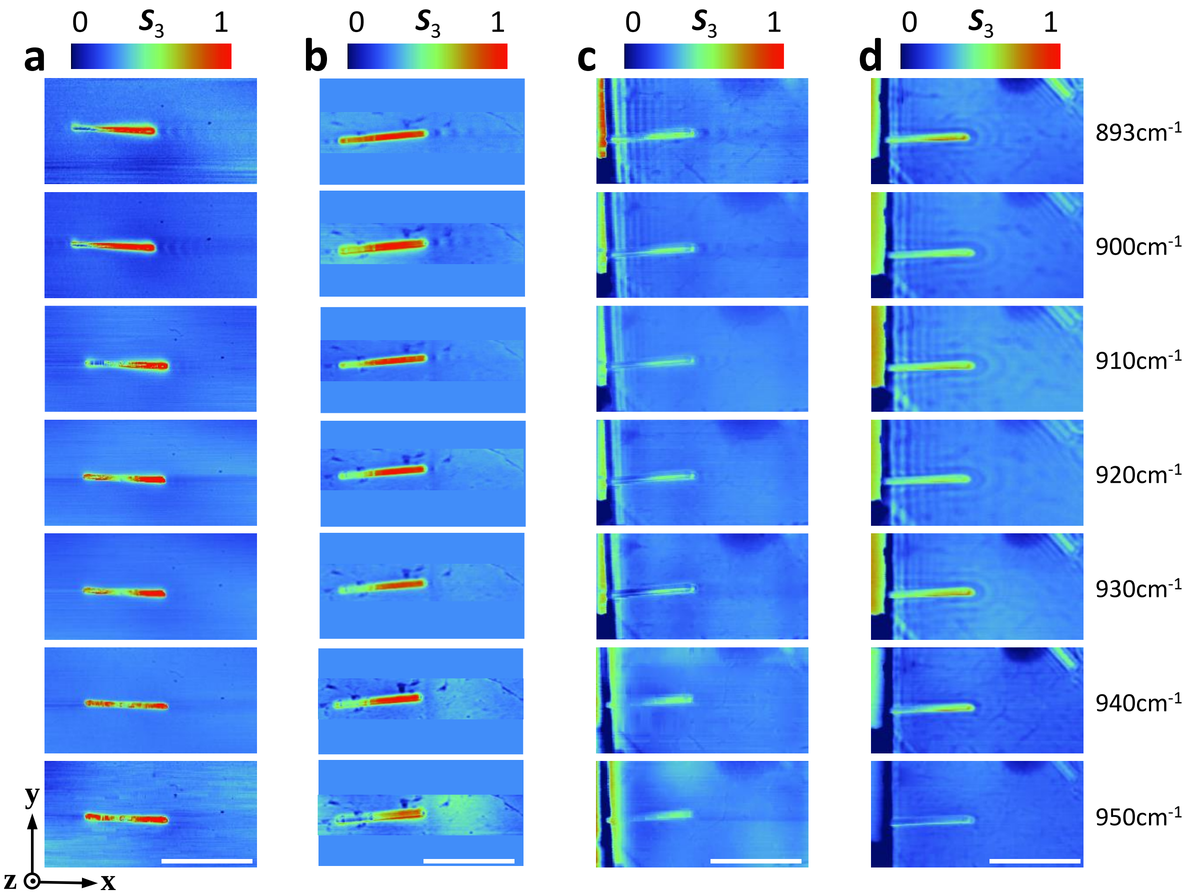

Supplementary Figure 8. Real-space infrared nanoimages reveal hybrid polaritons on the 60 nm Au substrate with different graphene Fermi energies and various illumination frequencies. (**a-d**) Antenna-launched hybrid polaritons measured in a heterostructure sample consisting of a 140-nm-thick α-MoO_3 film and monolayer graphene. The graphene Fermi energy is set at E_F=0 eV (**a**), E_F=0.3 eV (**b**), E_F=0.4 eV (**c**), and E_F=0.7 eV (**d**), respectively. The incident light wavelength is tuned from λ_0 = 11.20 μm (893 cm^{-1}) to 10.53 μm (950 cm^{-1}) in the experiments. The scale bars indicate 3 μm. Note that, we observe polariton fringes of two distinct periodicities (λ_p and $\lambda_p/2$) near the sample edge, the graphene boundary, and the edge of the gold antenna (d). The $\lambda_p/2$-period fringes can be assigned to the tip-launched polaritons reflected at the α-MoO3 edge or the graphene boundary. In addition, the λ_p-period fringes can be associated with antenna or edge launched polaritons propagating to the tip and being scattered to the detector[4,5].

Note that fringes near the sample edge reveal a decreased extra loss of hybrid polaritons with increasing doping (Supplementary Figures 7-8). This is because charged-impurity scattering leads to strongly reduced damping when increasing the carrier density, owing to the enhancement in electrostatic screening[6,7].

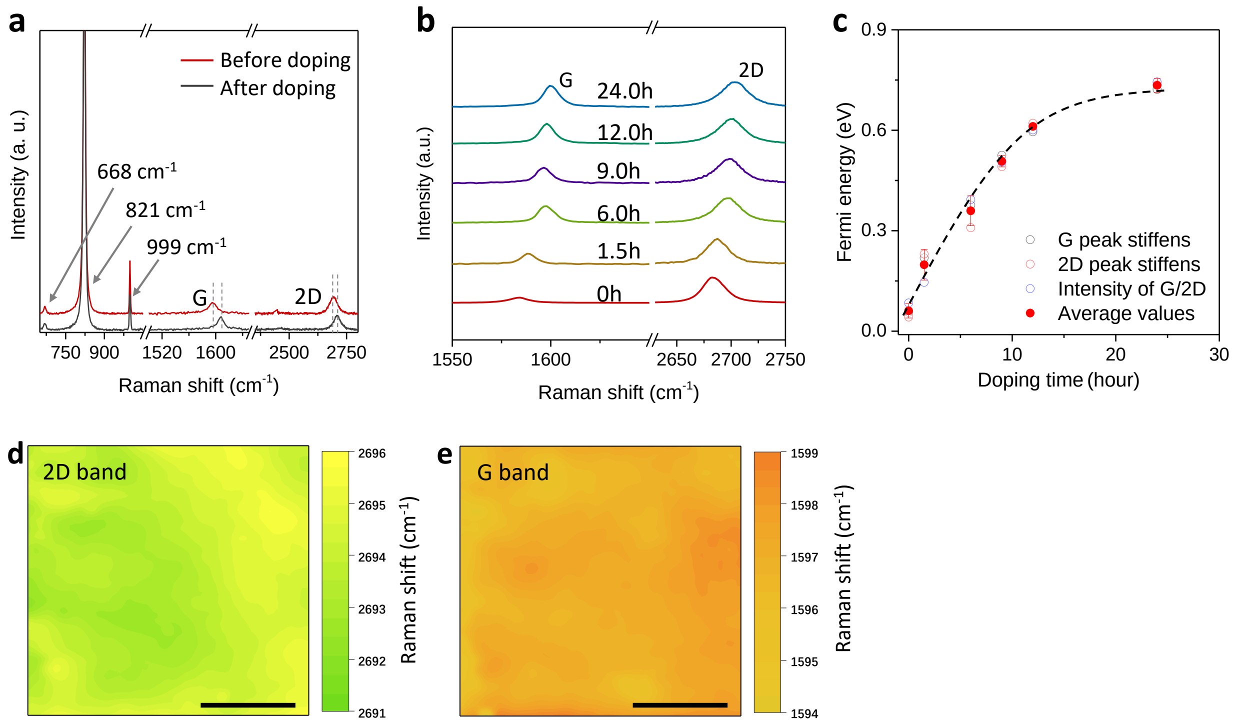

Supplementary Figure 9. Gas doping and Raman measurements of graphene samples. (a) Raman spectra of the G/α-MoO_3 heterostructure as obtained before and after doping of graphene. (**b**) Raman spectra of graphene after different doping times. The concentration of NO_2 gas is 75% in a N_2 atmosphere. **(c)** Fermi energies of graphene as a function of doping time. The Fermi energy is determined by following three different methods: G peak stiffening (grey circles), 2D peak stiffening (red circles), or the intensity ratio I_G/I_{2D} (blue circles). The solid red circles represent average values of the above three methods. Symbols are obtained from experimental data in (b), whereas the dashed curve is a guide to the eye. The sample used here has not been annealed in vacuum, and therefore, it shows a finite initial doping of ~ 0.1 eV. (**d, e**) Raman mapping of the peak position of 2D (d) and G (e) bands following gas doping of our graphene sample. Data in both plots show a uniformly distributed color with only a few localized spots, thus confirming that our sample has a rather uniform doping distribution.

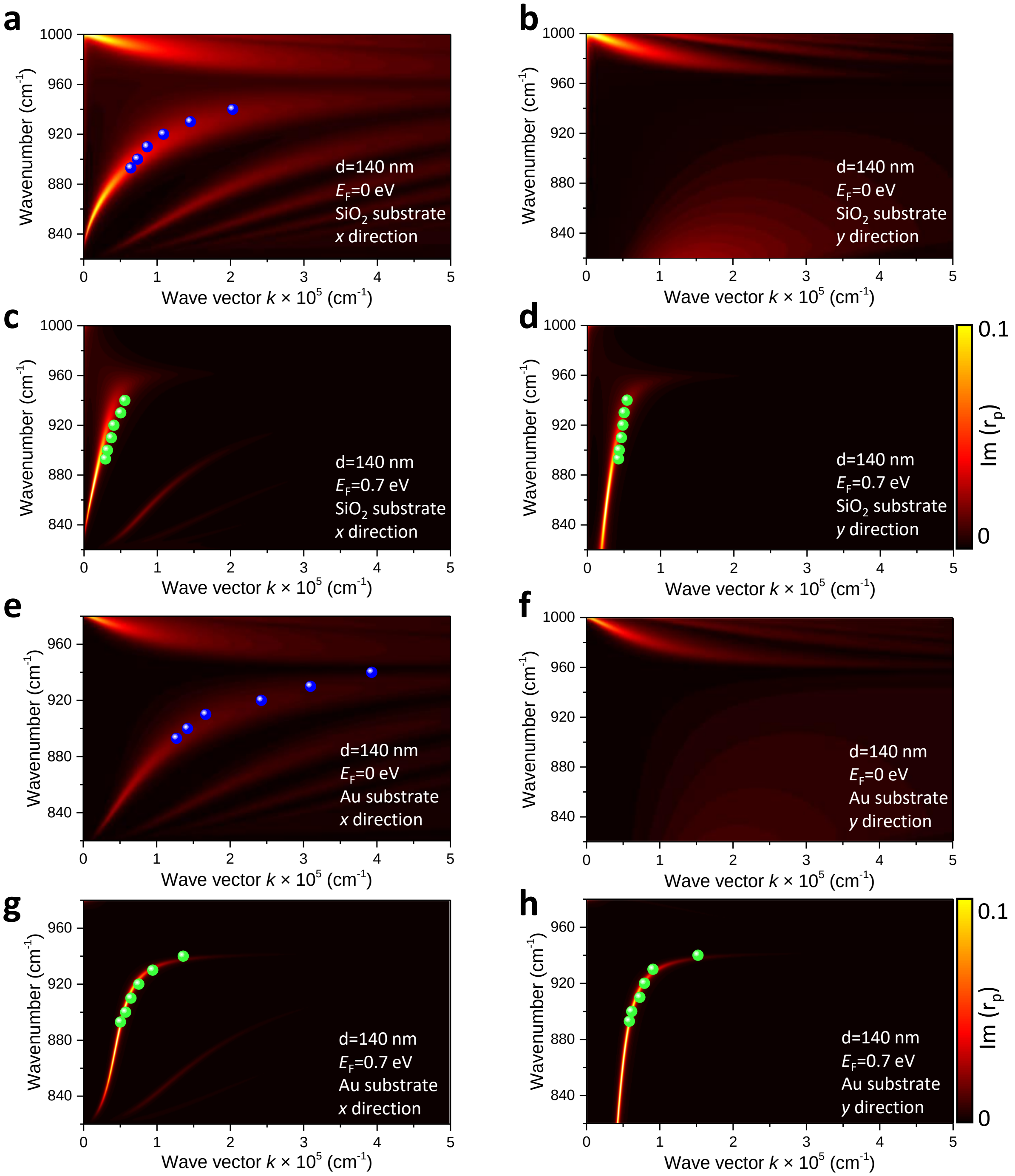

Supplementary Figure 10. Dispersions of hybrid polaritons. Theoretically calculated (color plots) dispersions of hybrid polaritons on the SiO_2 (a-d) or Au (e-h) substrates for wave vector along *x* ([100] direction of α-MoO_3, a, c, e, and g) and *y* ([001] direction of α-MoO_3, b, d, f, and h) with E_F=0 eV (a, b, e, and f) and E_F=0.7 eV (c, d, g, and h), respectively. Colored symbols are experimental data points extracted from Supplementary Figures 7 and 8.

When graphene is undoped (Supplementary Figures 10a, b), we find dispersion diagrams similar to those of PhPs supported in α-MoO_3 on SiO_2 substrate, where an intrinsic forbidden band (840-950 cm^{-1}) for polaritons propagating along the *y* direction can be seen in Supplementary Figure 10b, which leads to a hyperbolic isofrequency dispersion contour within the band. When graphene is highly doped (E_F=0.7 eV, Supplementary Figures 10c, d), the in-plane hybrid polariton is dominated by a graphene-plasmon character, where a bright feature with a steeper slope appears in the dispersion diagram in both propagating directions. As for the samples on the gold substrate (Supplementary Figures 10e-h), the hybrid polaritons become more subwavelength due to screening.

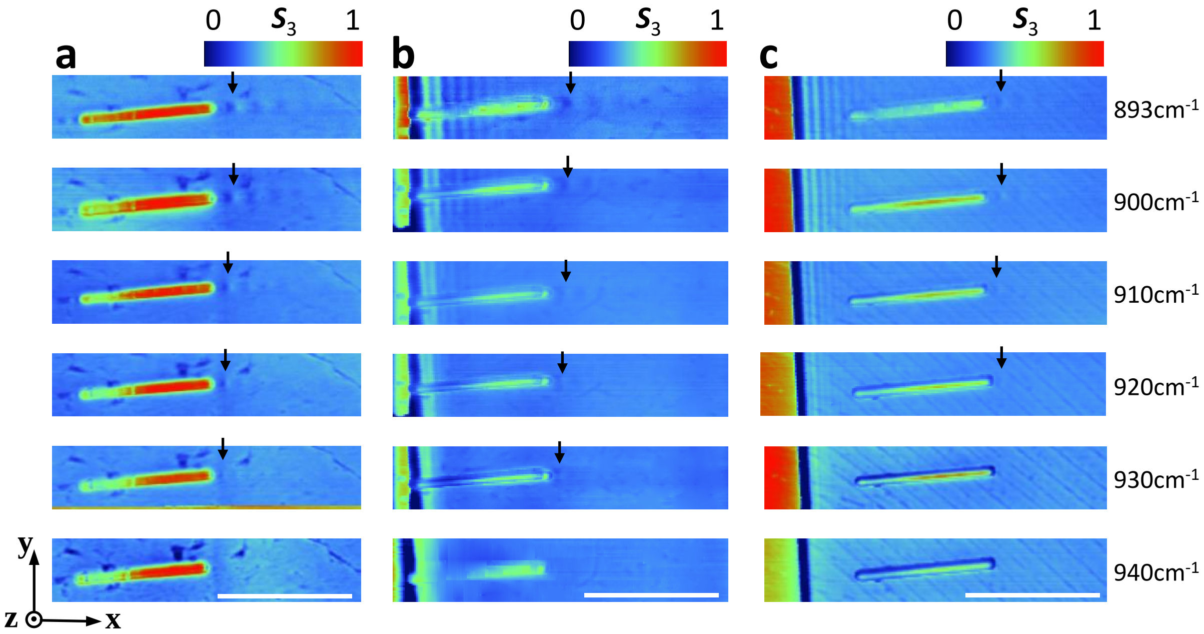

Supplementary Figure 11. Polariton canalizations near the topological transition at different illumination frequencies. (**a**) Images obtained from a sample with the graphene Fermi energy set at E_F=0.3 eV, an α-MoO_3 thickness of 140 nm, a substrate consisting of a 60-nm-thick gold substrate. (**b**) Same as (a), but for a graphene Fermi energy E_F=0.4 eV. (**c**) Same as (b), but for an α-MoO_3 thickness of 120 nm. The scale bars indicate 3 μm.

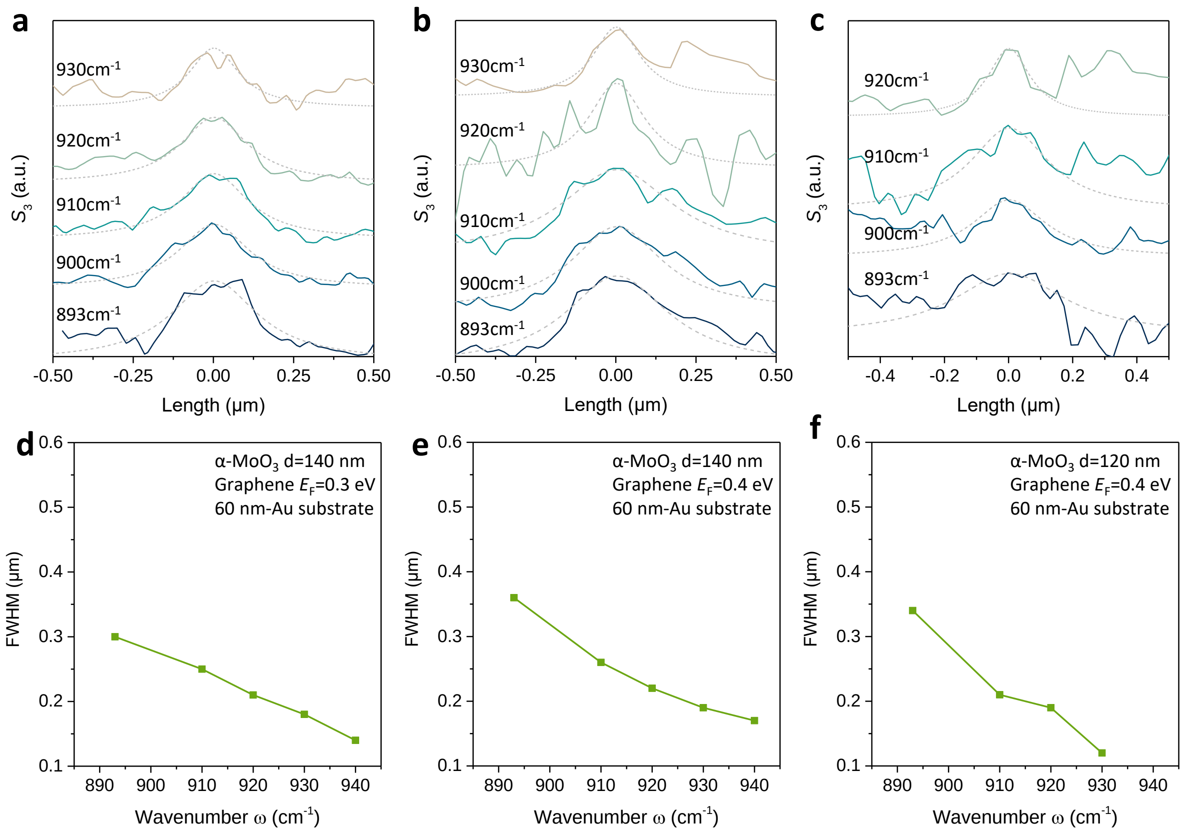

Supplementary Figure 12. The full width at half maximum (FWHM) of polariton canalizations. (**a-c**) Experimental near-field intensity (solid curves) at the black arrows in Supplementary Figures 11a-c, respectively. The dashed curves show Gaussian fittings of the experimental data. (**d-f**) Dependence of the FWHM of the spot size for polariton canalization as a function of incident light wavenumber ω for samples with different α-MoO_3 thickness and graphene Fermi energy (see labels). Green curves serve as a guide to the eye.

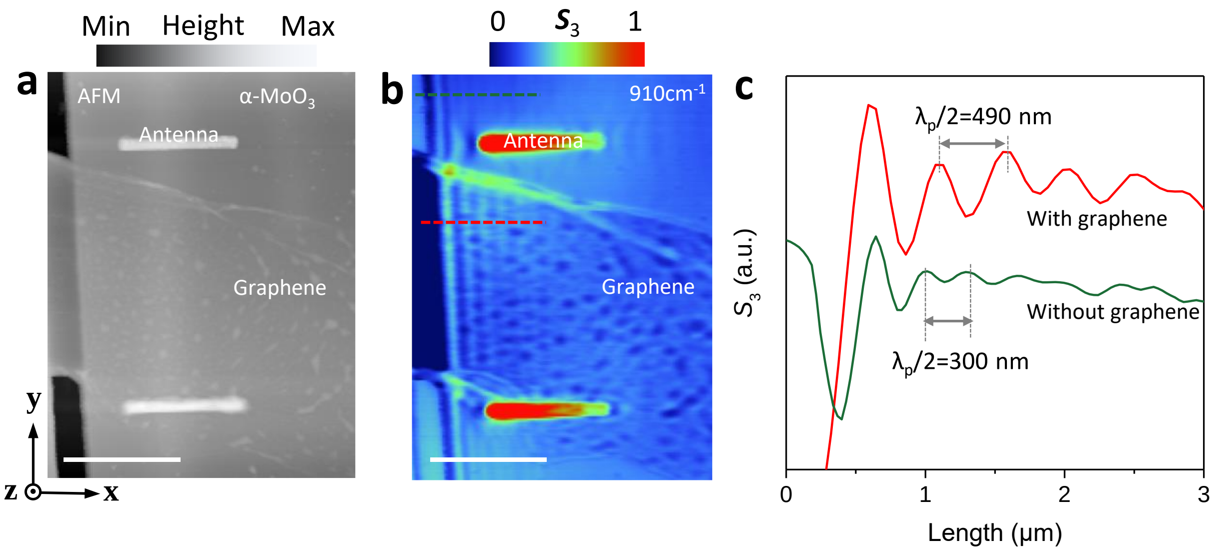

Supplementary Figure 13. Real-space infrared nanoimages reveal hybrid polaritons in a sample with disorder. (**a**) Topography image showing a graphene monolayer covering half of the α-MoO_3 region. (**b**) Near-field image recorded simultaneously with the topography shown in (a). The graphene Fermi energy is set at E_F=0.7 eV, and the α-MoO_3 thickness is 300 nm. The incident light wavelength is λ_0 = 10.99 μm (910 cm^{-1}). The scale bar indicates 3 μm. (**c**) Near-field profiles taken at the positions marked by green and red dashed lines in (b). The near-field data are shown in arbitrary units.

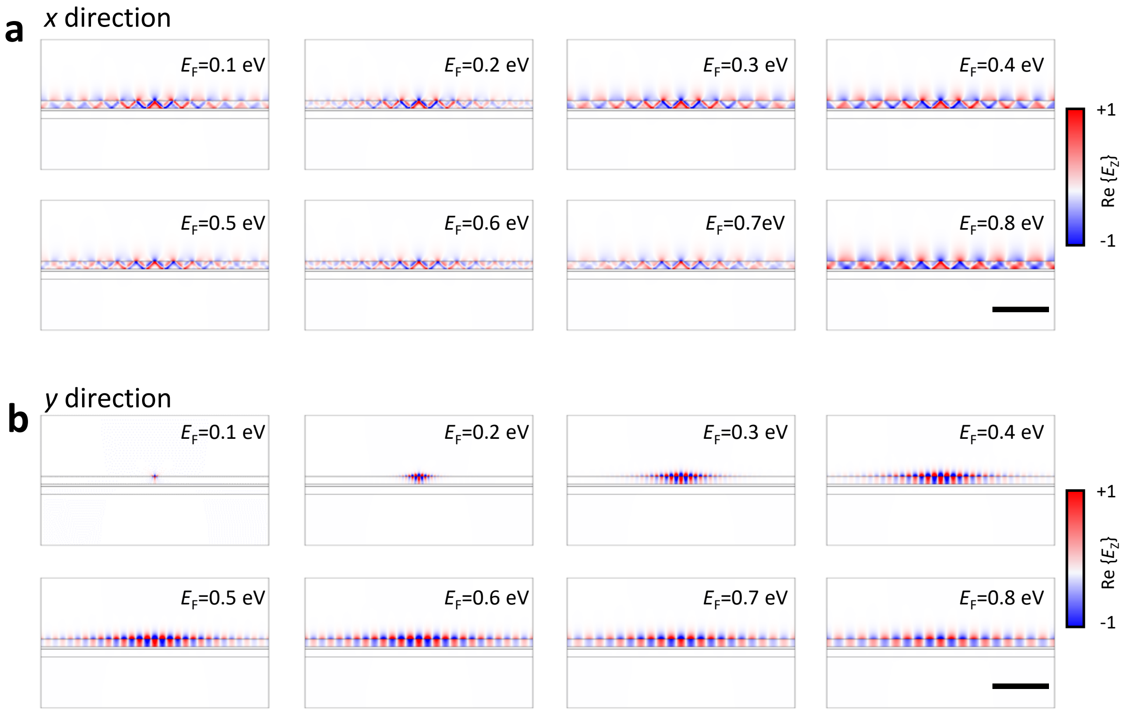

Supplementary Figure 14. Numerically simulated field distribution of the *z* component of hybrid polaritons at different Fermi energies of graphene along with *x* (a) and *y* in-plane directions (b). The sample is on a 60 nm gold substrate. A dipole is placed 100 nm above the graphene to launch the polaritons. The incident light wavelength is fixed at $\lambda_0 = 11.11$ μm (900 cm^{-1}) in all simulations.

Note 1. Mode analysis of hybrid polaritons from the coupling between graphene plasmons and phonon polaritons in α-MoO3.

Rearranging and simplifying Eq. (S8) in the main text, we can obtain the following transcendental equation:

1) For $\epsilon_t^{(2)} < 0$, i.e., $|\tan(\theta)| < \sqrt{-\frac{\epsilon_{xx}}{\epsilon_{yy}}}$,

$$\sqrt{\frac{\epsilon_t^{(2)}}{\epsilon_z^{(2)}}}\sqrt{\varepsilon_z^{(2)}\frac{\omega^2}{c^2} - q^2}d_h = \tan^{-1}\left(\frac{\sqrt{\epsilon_t^{(2)}\epsilon_z^{(2)}}\sqrt{q^2 - \epsilon_a\frac{\omega^2}{c^2}}}{\epsilon_a\sqrt{\varepsilon_z^{(2)}\frac{\omega^2}{c^2} - q^2}\left(1 + \frac{i\sigma Z_0\sqrt{q^2 - \epsilon_a\frac{\omega^2}{c^2}}}{\epsilon_a\omega}\right)}\right)$$

$$+\tan^{-1}\left(\frac{\sqrt{\epsilon_t^{(2)}\epsilon_z^{(2)}}\sqrt{q^2-\epsilon_s\frac{\omega^2}{c^2}}}{\epsilon_s\sqrt{\varepsilon_z^{(2)}\frac{\omega^2}{c^2}-q^2}}\right)+\pi l \qquad (S9)$$

2) For $\epsilon_t^{(2)} > 0$, i.e., $|\tan(\theta)| > \sqrt{-\frac{\epsilon_{xx}}{\epsilon_{yy}}}$,

$$-2i\sqrt{\frac{\epsilon_t^{(2)}}{\epsilon_z^{(2)}}}\sqrt{\varepsilon_z^{(2)}\frac{\omega^2}{c^2}-q^2}d_h = \ln\left(\frac{1-\frac{\sqrt{\epsilon_t^{(2)}\epsilon_z^{(2)}}\sqrt{\epsilon_a\frac{\omega^2}{c^2}-q^2}}{\epsilon_a\sqrt{\varepsilon_z^{(2)}\frac{\omega^2}{c^2}-q^2}}+\frac{\sigma Z_0\sqrt{\epsilon_a\frac{\omega^2}{c^2}-q^2}}{\omega\epsilon_a}}{1+\frac{\sqrt{\epsilon_t^{(2)}\epsilon_z^{(2)}}\sqrt{\epsilon_a\frac{\omega^2}{c^2}-q^2}}{\epsilon_a\sqrt{\varepsilon_z^{(2)}\frac{\omega^2}{c^2}-q^2}}+\frac{\sigma Z_0\sqrt{\epsilon_a\frac{\omega^2}{c^2}-q^2}}{\omega\epsilon_a}}\right)$$

$$+\ln\left(\frac{1-\frac{\sqrt{\epsilon_t^{(2)}\epsilon_z^{(2)}}\sqrt{\epsilon_s\frac{\omega^2}{c^2}-q^2}}{\epsilon_s\sqrt{\varepsilon_z^{(2)}\frac{\omega^2}{c^2}-q^2}}}{1+\frac{\sqrt{\epsilon_t^{(2)}\epsilon_z^{(2)}}\sqrt{\epsilon_s\frac{\omega^2}{c^2}-q^2}}{\epsilon_s\sqrt{\varepsilon_z^{(2)}\frac{\omega^2}{c^2}-q^2}}}\right) \qquad (S10)$$

Here, l is an integer that labels different branches. Solving Eqs. (S9) and (S10) at a fixed frequency, we can obtain the IFCs shown in Supplementary Figure 2.

For $|\tan(\theta)| < \sqrt{-\frac{\epsilon_{xx}}{\epsilon_{yy}}}$, Eq. (S9) suggests that the propagating polariton is a volume mode and most of the energy is confined inside the α-MoO_3 film. Graphene is used to tune the wavelength of hybrid polaritons by changing the phase of the waveguide (as shown in the second term in Eq. (S9)). Supplementary Figure 14a shows a zig-zag propagation mode in the α-MoO_3 film, which is consistent with phonon polaritons in α-MoO_3. For $|\tan(\theta)| > \sqrt{-\frac{\epsilon_{xx}}{\epsilon_{yy}}}$, the in-plane dielectric function of α-MoO_3 is positive, which can be regarded as a dielectric substrate for graphene plasmons. In this situation, most of the mode energy is

confined at the interface between the graphene layer and the α-MoO_3 film, while the electric field in the latter is evanescent. Supplementary Figure 14b shows a surface mode in the graphene layer, whose wavelength increases with the doping level, which is a behavior similar to graphene plasmons on a SiO_2 substrate.

Along the *y* direction, the mode is always dominated by graphene plasmons, as long as the contour has a closed shape. As the doping level is further increased, the graphene plasmon wavelength becomes larger, thus resulting in a smaller wave vector along the *y* direction. As for the mode propagating along the *x* direction, it is dominated by the α-MoO_3 phonon polaritons (PhPs). Changing the graphene doping level can be considered as varying the dielectric environment for the α-MoO_3 phonon polaritons.

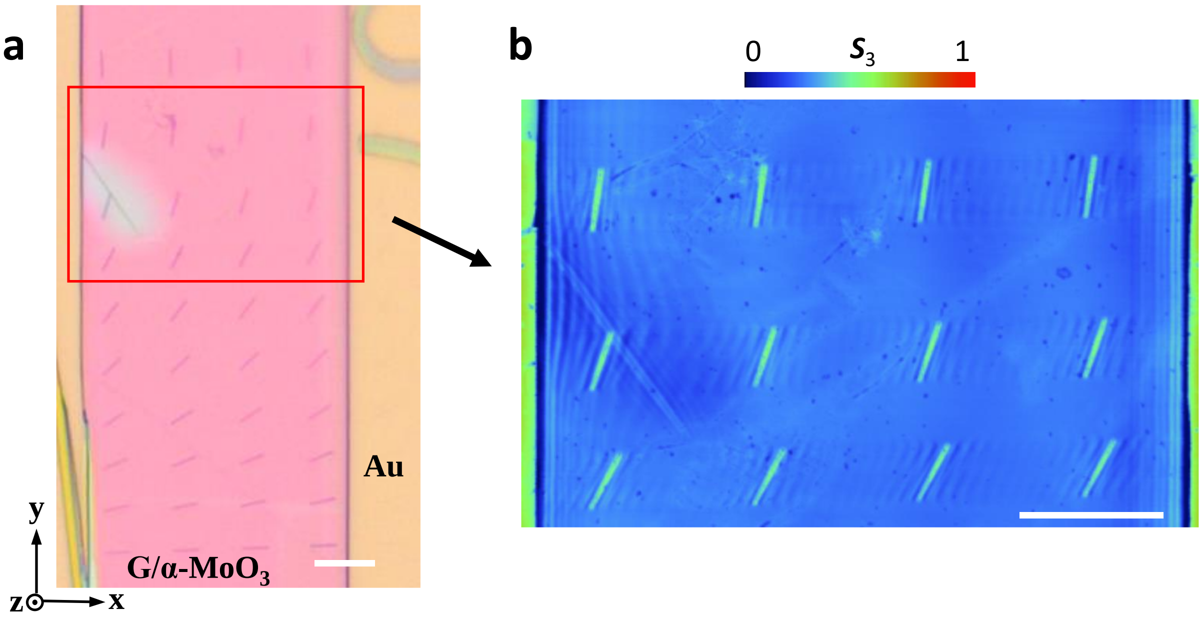

Supplementary Figure 15. Near-field imaging of antenna-launched polaritons in a sample with four sets of repeated antenna arrays. (a) Optical image of a graphene/α-MoO_3 heterostructure on a gold substrate with different angles of the antenna. The α-MoO_3 thickness is 207 nm. **(b)** Near-field nanoimaging of the antenna areas is highlighted by the red rectangle in panel (a). There are four sets of repeated antennas fabricated on the sample, and we scan the four antenna arrays with the same rotation angle in one single near-field measurement. Then, we can extract the average as well as error bars for the polariton wave vectors, which are shown in Figures 3e and 3f of the main text. The scales bars indicate 6 μm.

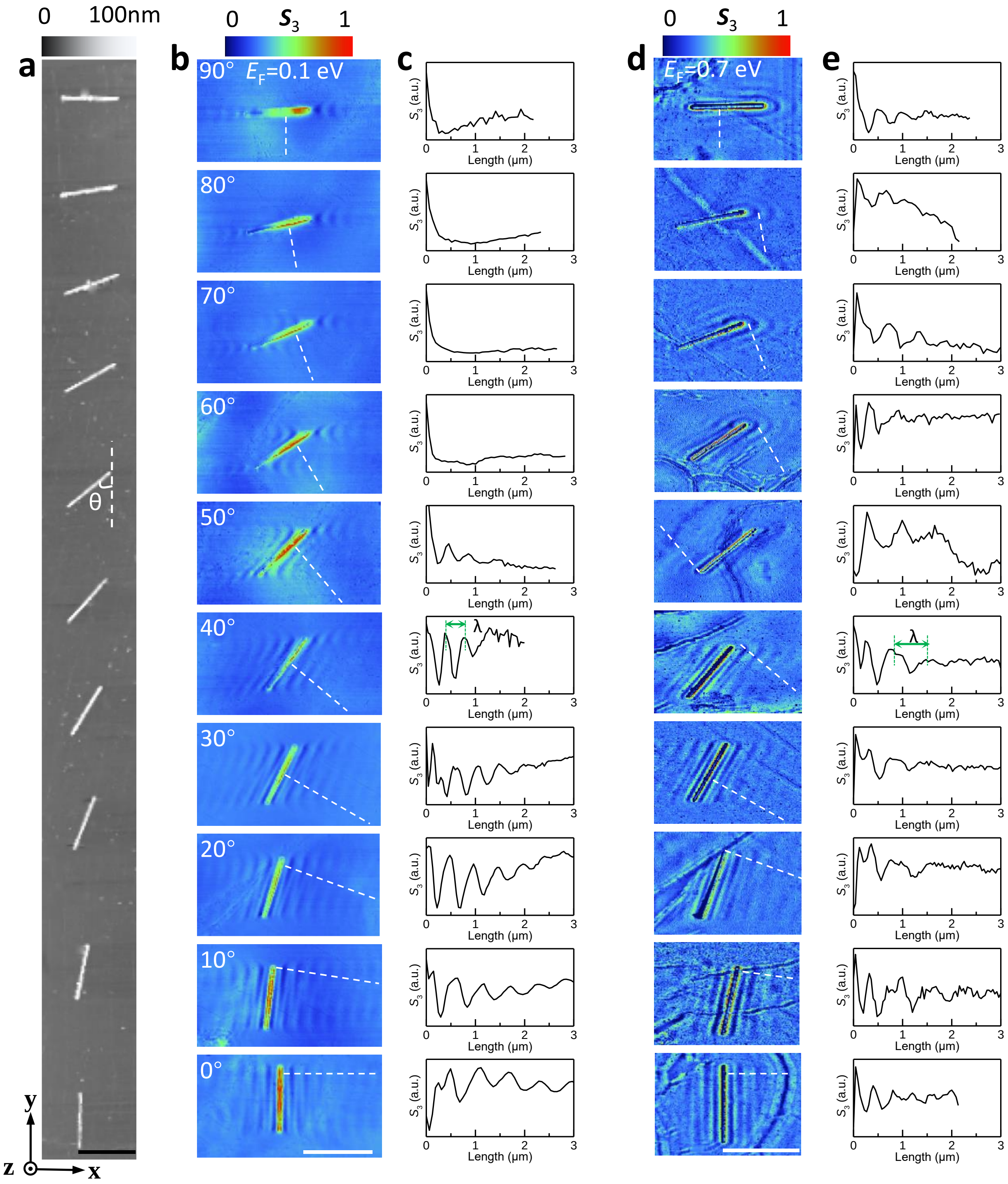

Supplementary Figure 16. Extraction analysis of antenna-tailored launching of hybrid polaritons. (a) Topography image of a graphene/α-MoO_3 sample on a gold substrate with various gold antennas oriented along different rotation angles θ with respect to the *y* direction. **(b)** Experimentally measured near-field amplitude images of hybrid polaritons launched by gold antennas with rotation angle θ ranging from 0 to 90°.

The graphene Fermi energy is set to E_F=0.1 eV. **(c)** Near-field intensity along the white dashed lines in panel (b). The difference in phase between the two ends would cause some trouble in extracting the wave vector. However, these interferences are mainly placed in the middle of the antenna, and our valid data are all taken at the endpoints of the antenna, with very limited influence from the phase shift. **(d)** Same experimental measurements as in panel (b), but with the graphene Fermi energy set to E_F=0.7 eV. The artifacts of graphene in panel (d) compared to (b) originate in grain boundaries. Such grain boundaries are barely observable in the near-field nanoimage of undoped graphene. However, when the graphene is highly doped, surface plasmons are reflected and scattered by the grain boundaries, thus causing plasmon interference[8]. **(e)** Near-field intensity along the white dashed lines in panel (d). The incident light wavelength is fixed at λ_0 = 11.11 μm (900 cm^{-1}). The scale bar indicates 3 μm.

Note that the launching efficiency should decrease with decreasing in-plane rotation angle θ due to the associated change in the coupling efficiency between the antenna and the external illumination. In spite of this, the launched-polariton fringes are already sufficiently clear for us to extract the wave vector in the experiments.

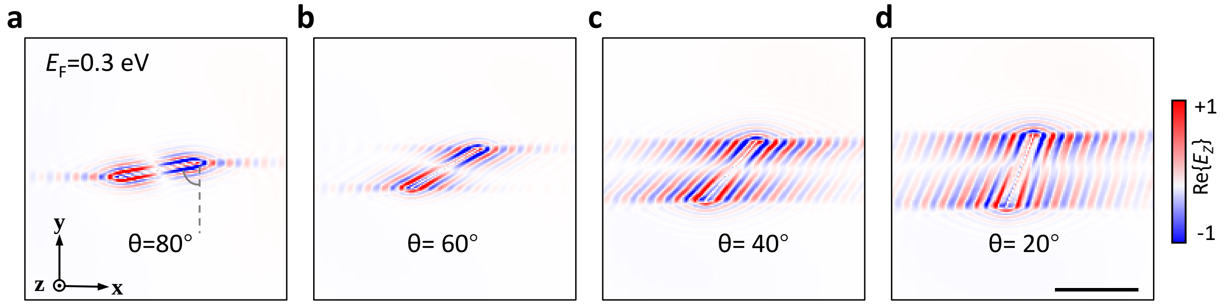

Supplementary Figure 17. Numerically simulated field distribution of hybrid polaritons launched by gold antennas with different antenna angles. The graphene Fermi energy is set at E_F=0.3 eV. The thickness of α-MoO_3 is 207 nm. The scale bar indicates 3 μm. The incident light wavelength is fixed at λ_0 = 11.11 μm (900 cm^{-1}).

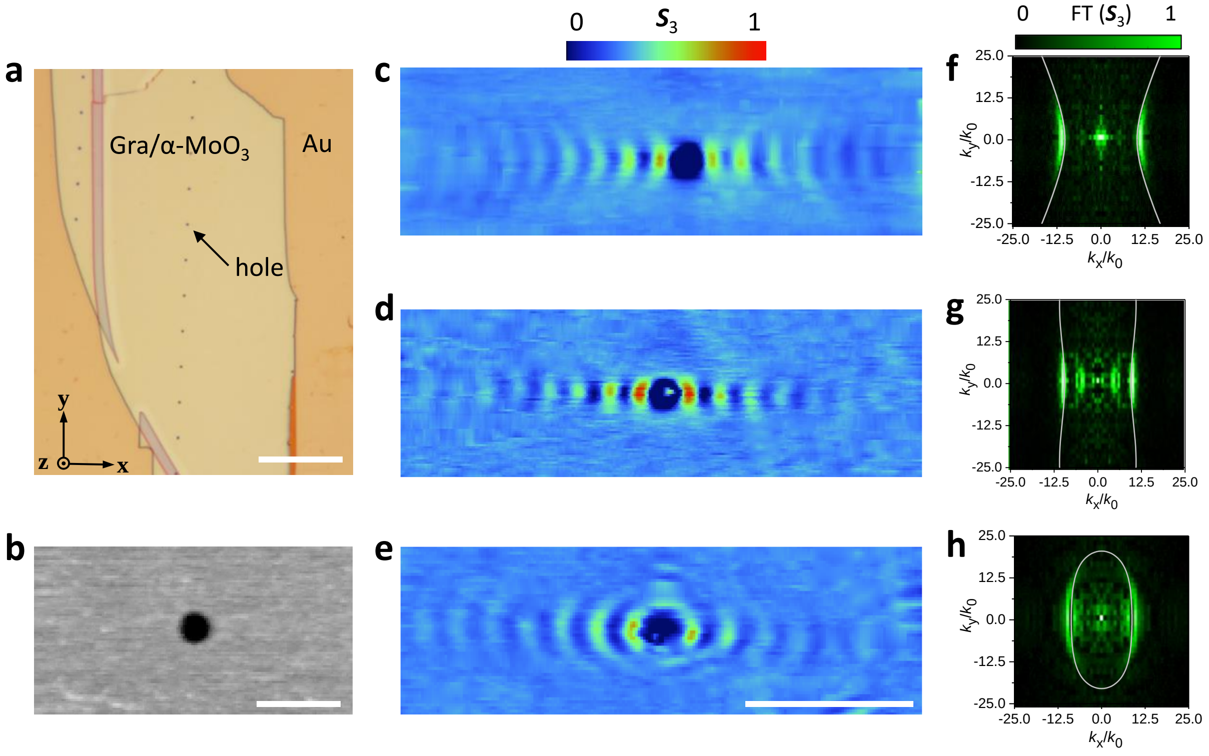

Supplementary Figure 18. Reconstruction of isofrequency contours by launching hybrid polaritons in samples with defects. To avoid the effects of the antenna rotation angle, we prepare a sample with defects that allow us to resolve the wave vectors in all directions at once, from a single scan of the near-field**. (a)** Optical image of a graphene/α-MoO_3 sample with fabricated circular holes of 400 nm diameter. The thickness of the α-MoO_3 film is 305 nm. The scale bar indicates 5 μm. **(b)** AFM image of the hole. The scale bar indicates 1.2 μm. **(c-e)** Experimentally measured near-field amplitude images of hybrid polaritons launched by the AFM tip and reflected by the defects. The incident light wavelength is fixed at λ_0 = 11.11 μm (900 cm^{-1}). The scale bar indicates 3 μm. (**f-h**) Absolute value of the spatial Fourier transforms of the experimental near-field images shown in panels (c-e), revealing the isofrequency contours of hybrid polaritons. The grey curves represent theoretically calculated isofrequency contours.

Note that in this sample, polaritons are excited by the tip in all directions with similar launching efficiency, and they are reflected back by the defects. Our experimental results and calculations for this sample are in excellent agreement as well.

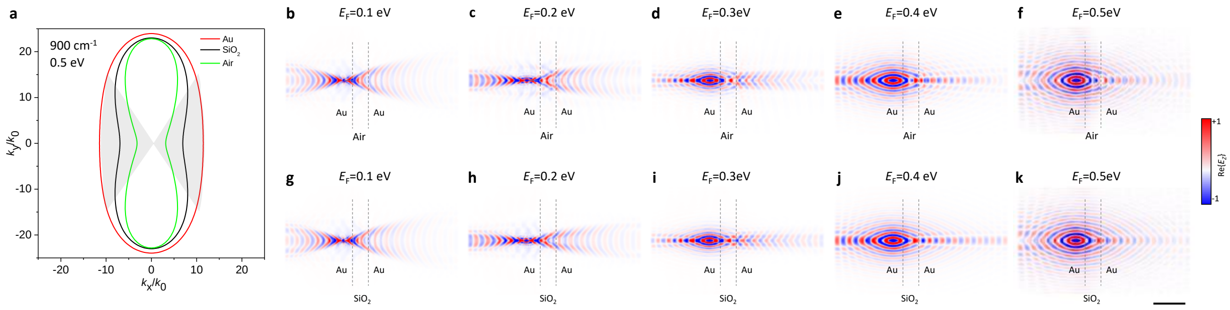

Supplementary Figure 19. **Numerically simulated partial focusing of hybrid polaritons based on SiO_2 and air lenses with different Fermi energies of graphene from $E_F = 0.1$ to 0.5 eV.** (**a**) Isofrequency dispersion contours of hybrid polaritons for Au (red), SiO_2 (black), and air (green) substrates at 900 cm^{-1} ($\lambda_0 = 11.11$ μm). The shaded areas highlight convex and concave dispersion contours in the region around the *x* axis on the gold and SiO_2 substrates, where the dispersion contour of polaritons on the air substrate is also concave. The IFC of polaritons on suspended graphene/α-MoO_3 is also concave around the *x* axis and very similar to that on the SiO_2 substrate, since the dielectric constants of both air and SiO_2 are much smaller than that of gold at 900 cm^{-1}. (**b-k**) Hybrid polaritons excited at a point placed 900 nm to the left of the edge in the left Au substrate and propagating first directionally towards the air (**b-f**) or SiO_2 (**g-k**) lens and later to the right Au area. Vertical dashed lines represent the interface. The width of the SiO_2 and air lenses is 1.5 μm. The scale bar indicates 3 μm. The incident light wavelength is fixed at λ_0 = 11.11 μm (900 cm^{-1}).

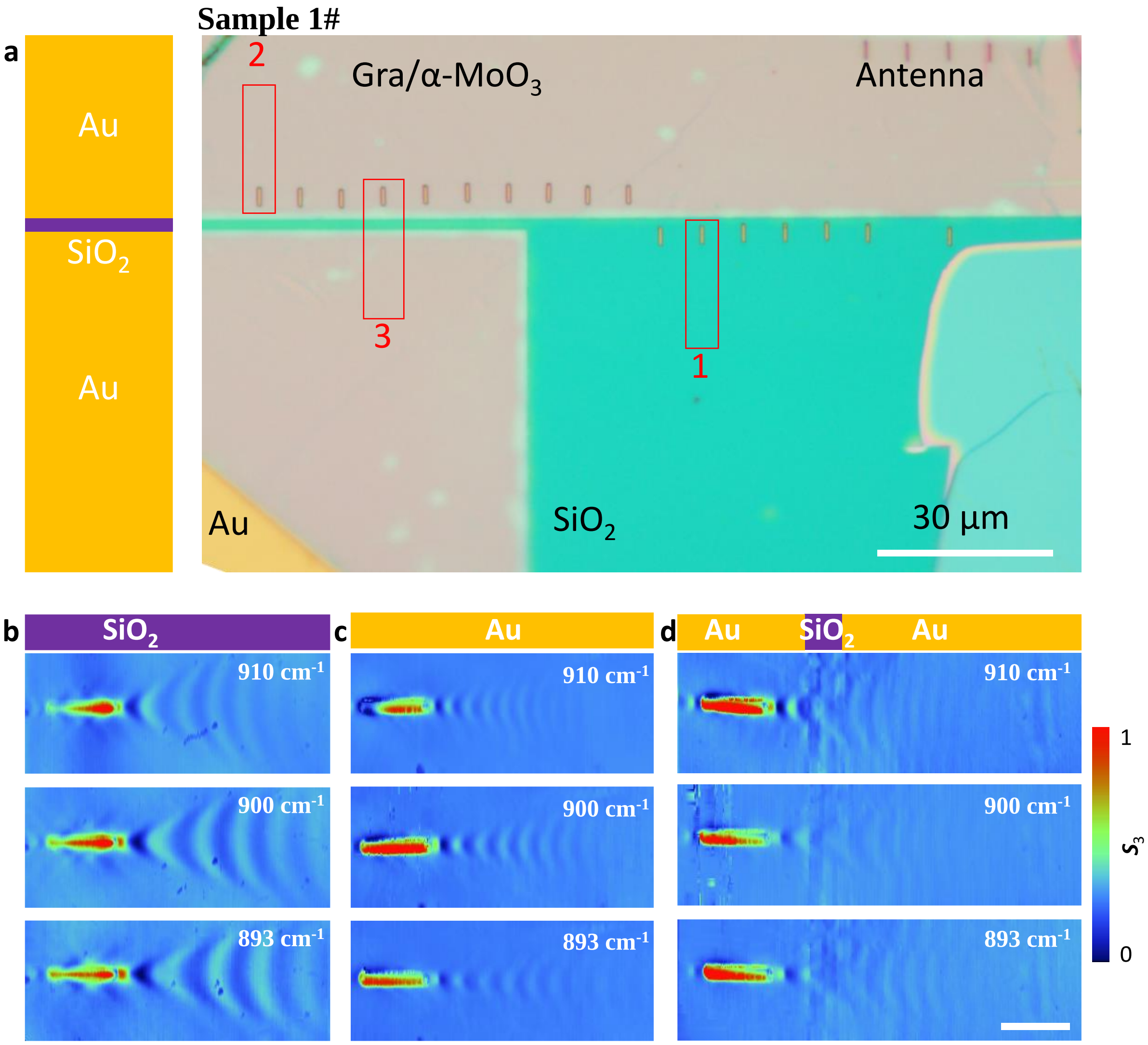

Supplementary Figure 20. Near-field characterization of graphene/α-MoO_3 on the Au-SiO_2-Au in-plane sandwich structure before doping. (a) Optical overview image of the sample (numbered 1#, with sample areas numbered 1, 2, 3). The thickness of α-MoO3 is 320 nm. The scale bar indicates 30 μm. (**b-d**) Experimentally measured polariton near-field distributions for areas on SiO_2 (b), gold (c), and Au-SiO_2-Au (d) substrates, respectively, subject to different illumination frequencies at 893 cm^{-1}, 900 cm^{-1}, and 910 cm^{-1}, respectively. Panels (b), (c), and (d) correspond to areas 1, 2, and 3 in panel (a), respectively. The graphene Fermi energy is $E_F = 0.1$ eV. The scale bar indicates 3 μm. The horizontal orange and purple stripes on the upper part of the near-field images indicate the gold and SiO_2 substrates.

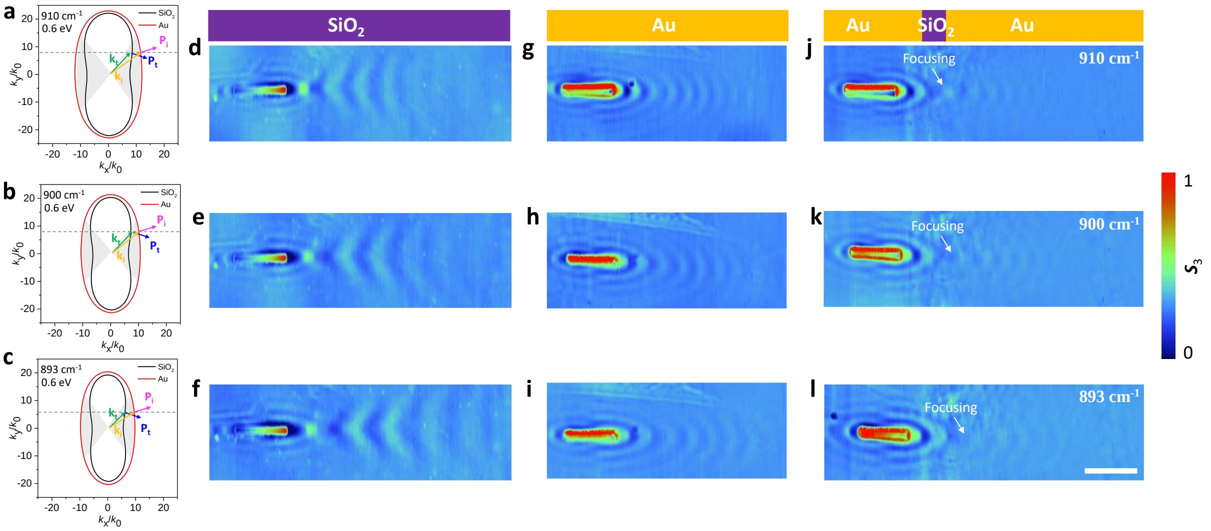

Supplementary Figure 21. Partial focusing of the sample in Supplementary Figure 20a at E_F=0.6 eV. (a-c) Isofrequency dispersion contours of hybrid polaritons for Au (red) and SiO_2 (black) substrates with a graphene Fermi energy of 0.6 eV for different illumination frequencies set at 893 cm^{-1}, 900 cm^{-1}, and 910 cm^{-1}, respectively. The shaded areas highlight convex and concave dispersion contours in the region around the *x* axis on the gold and SiO_2 substrates, respectively. **(d-l)** Experimentally measured polariton near-field distributions for samples on SiO_2 (d-f), gold (g-i), and Au-SiO_2-Au (j-l) substrates, respectively, for different illumination frequencies. Panels (d-f), (g-i), (j-l) correspond to the areas labeled 1, 2, 3 of sample 1# in Supplementary Figure 20a, respectively. The graphene Fermi energy is E_F=0.6 eV in all cases. The thickness of α-MoO_3 is 320 nm. The scale bar indicates 3 μm. The horizontal orange and purple stripes on the upper part of near-field images indicate the gold and SiO_2 substrates.

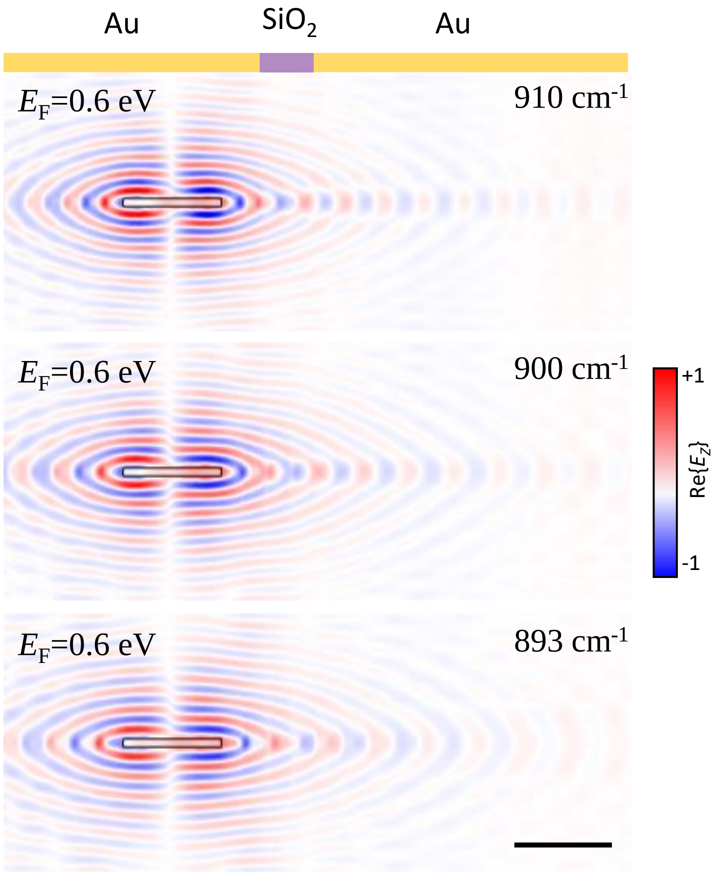

Supplementary Figure 22. Numerically simulated field distributions of partial focusing. We show Re{E_z} corresponding to the experiments shown in Figure 4c and Supplementary Figure 21j-l. The field is evaluated 50 nm above the surface of the heterostructure. The scale bar indicates 3 μm.

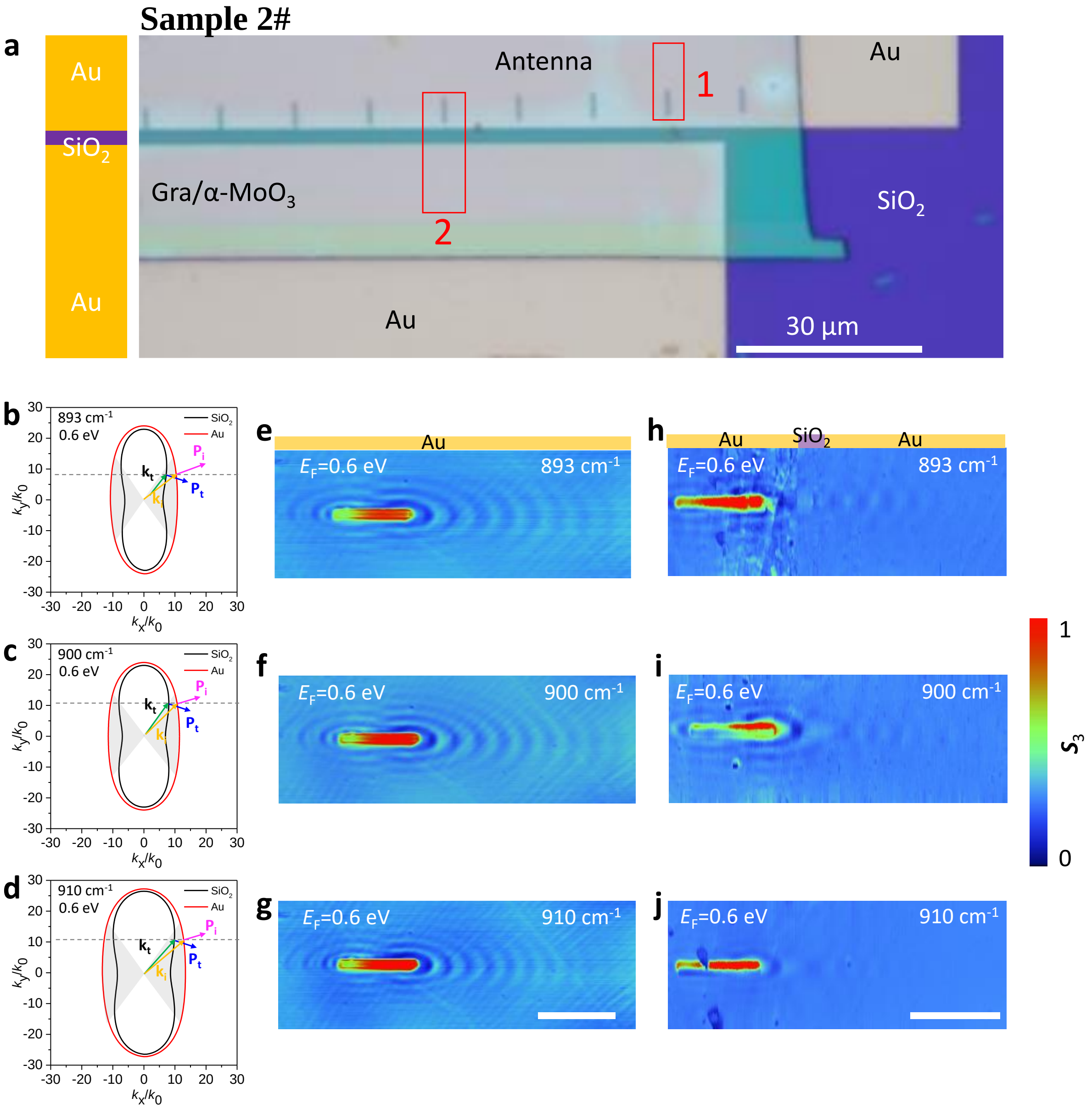

Supplementary Figure 23. Partial focusing of a sample at E_F=0.6 eV. (a) Optical images of the sample (numbered 2#, with sample areas numbered 1, 2). The scale bar indicates 30 μm. **(b-d)** Isofrequency dispersion contours of hybrid polaritons for Au and SiO_2 substrates at frequencies of 893 cm^{-1}, 900 cm^{-1}, and 910 cm^{-1} with E_F= 0.6 eV. The shaded areas highlight convex and concave dispersion contours in the region around the *x* axis on the gold and SiO_2 substrates, respectively. **(e-j)** Experimentally measured polariton near-field distributions for samples on gold (e-g) and Au-SiO_2-Au (h-j) substrates, respectively, for different illumination frequencies. Panels (e-g) and (h-j), corresponding to areas 1 and 2 in panel (a), respectively. The graphene Fermi energy is E_F=0.6 eV. The thickness of α-MoO_3 is 240 nm. The scale bars indicate 3 μm. The horizontal orange and purple stripes on the upper part of the near-field images indicate the gold and SiO_2 substrates.

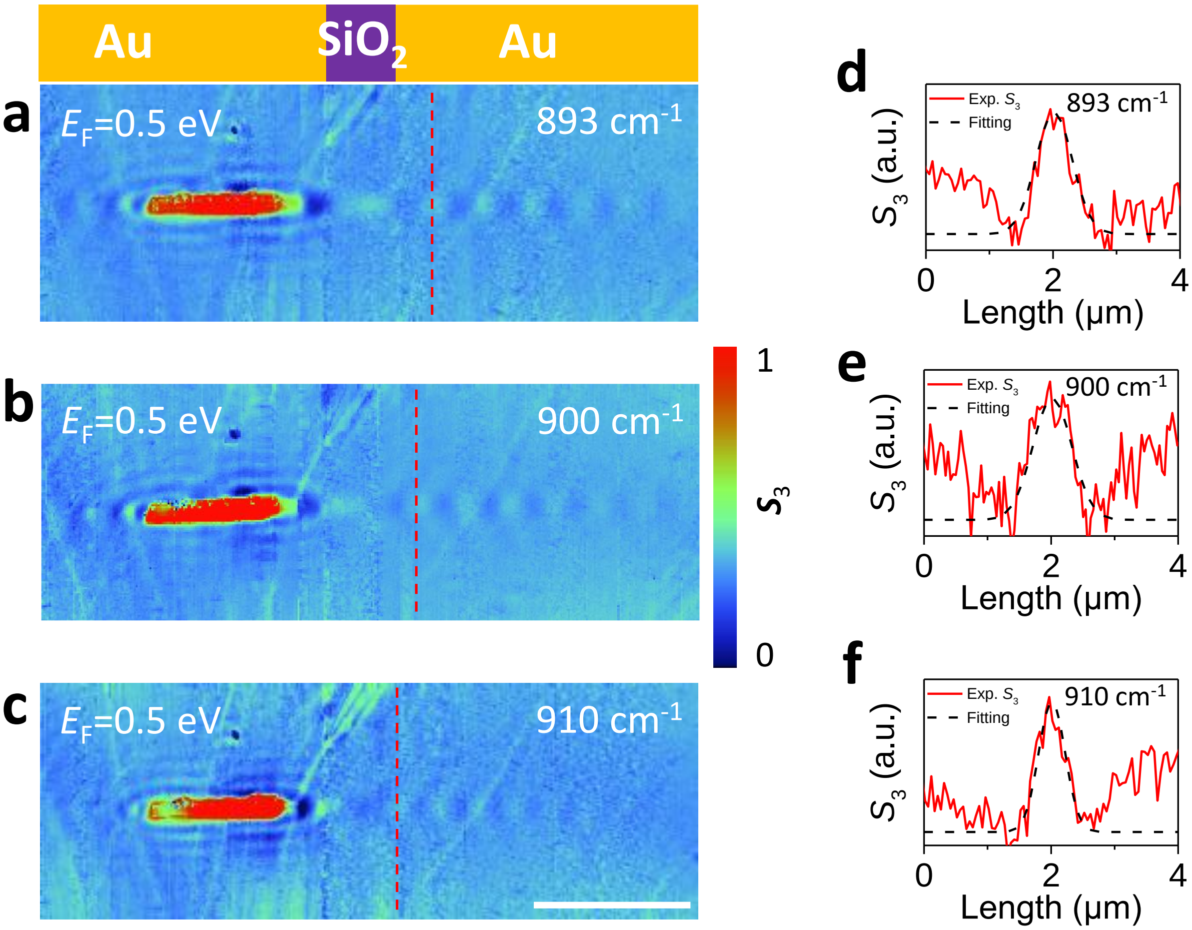

Supplementary Figure 24. Partial focusing of sample in Supplementary Figure 23 at E_F=0.5 eV. (a-c) Near-field image of antenna-launched hybrid polaritons, corresponding to areas 1 and 2 of sample 2# in Supplementary Figure 23a. The scale bar indicates 3 μm. The polaritons are launched by a gold antenna (length 3 μm, width 250 nm, thickness 50 nm). The α-MoO_3 film is 240 nm thick. The graphene Fermi energy is fixed at E_F= 0.5 eV. The horizontal orange and purple stripes on the upper part of the near-field images indicate the gold and SiO_2 substrates. (**d-f**) Near-field profiles taken at the positions marked by red vertical dashed lines in panels (a-c). The black dashed curves are Gaussian fittings. Note that, after propagating through such an image point, the focused polariton waves should propagate divergently. In our experiments, the canalization wavefront is due to the fact that the loss of hybrid polaritons along the *y* direction is much larger than that along the *x* direction (see the dispersion in Supplementary Figure 10). To observe the divergent propagation, one can further increase the doping of graphene and thus enhance plasmon propagation along the *y* direction (Figure 4 in the main text and Supplementary Figures 21 and 23).

Note that Supplementary Figures 23-24 discuss another sample to experimentally corroborate the effect of partial focusing. It is worth mentioning that, with fine control of

the graphene Fermi energy, partial focusing can further achieve diffraction-free propagation by means of the canalized wavefronts.

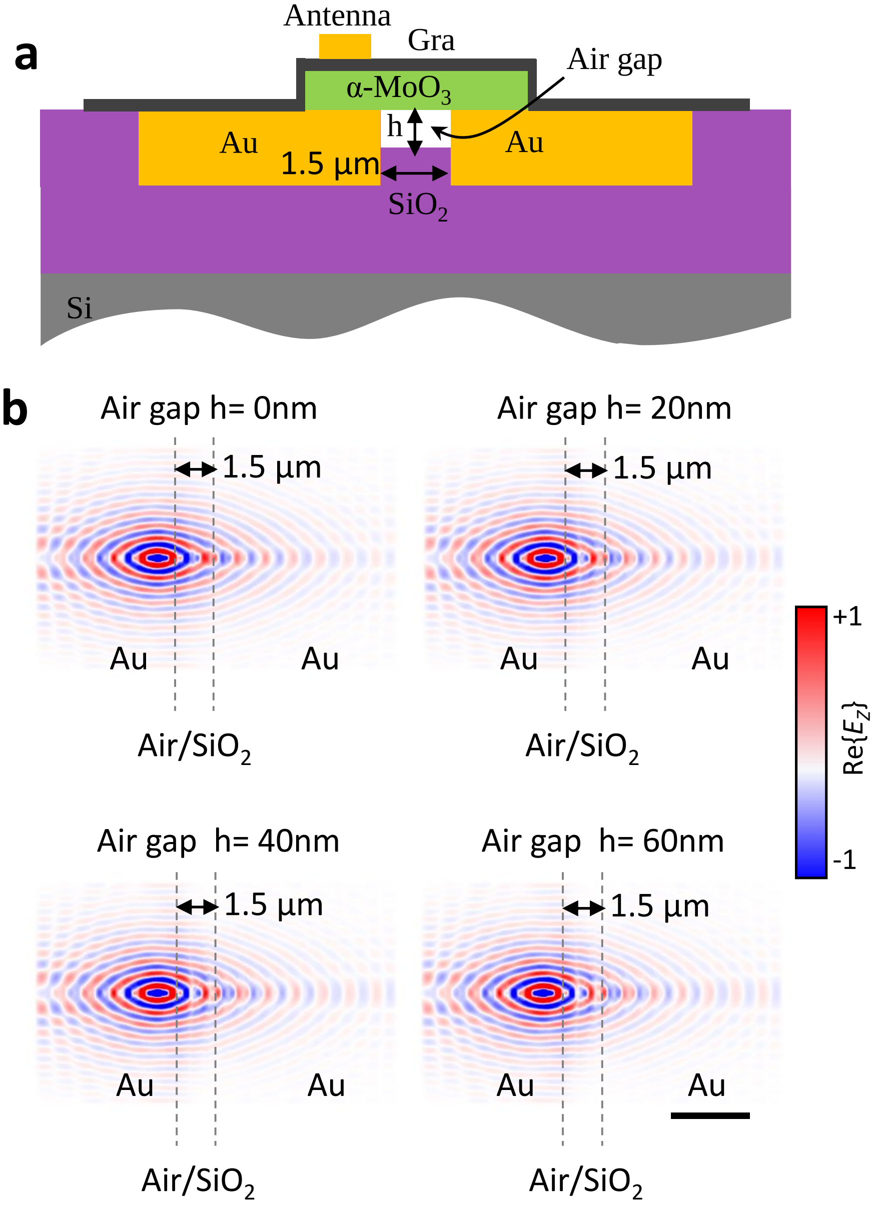

Supplementary Figure 25. Numerically simulated partial focusing of hybrid polaritons with different heights of the air gap from 0 to 60 nm. (**a**) Schematics of the Au-SiO_2-Au in-plane sandwich structure with an air gap of height *h*. **(b)** Numerically simulated field distribution of hybrid polaritons launched by a dipole placed 100 nm above the graphene. The hybrid polaritons are excited at a point placed 900 nm to the left of the edge of the Au substrate and propagate first directionally towards the air/SiO_2 area with different heights of the air gap, and later to the right Au area. The width of the SiO_2 lens is 1.5 µm. The scale bar indicates 3 µm. The incident light wavelength is fixed at λ_0 = 11.11 µm (900 cm^{-1}).

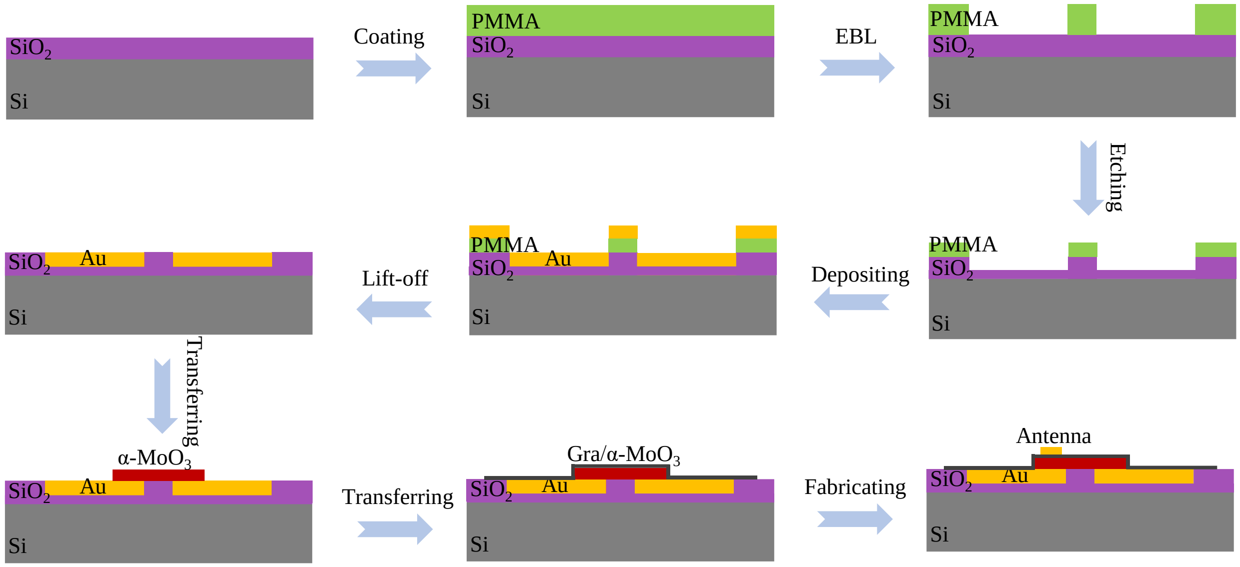

Supplementary Figure 26. Fabrication process of the in-plane Au-SiO_2-Au sandwich structure. The in-plane sandwich structures were patterned on a 300 nm SiO_2/500 µm Si substrate using 100 kV electron-beam lithography (EBL) (Vistec 5000+ES, Germany) on approximately 1 µm of PMMA resist. The patterns were then etched with C_4F_8 and SF_6 (North Microelectronics, DSE200). AFM imaging was used to monitor the etching depth. A Magnetron Sputtering Coating (Lab-18) setup was subsequently used to deposit a 55-nm-Au layer in a vacuum chamber at a pressure of $<5\times10^{-6}$ Torr to fabricate the in-plane Au-SiO_2-Au sandwich structure, followed by lift-off to remove any residual organic materials and Au.

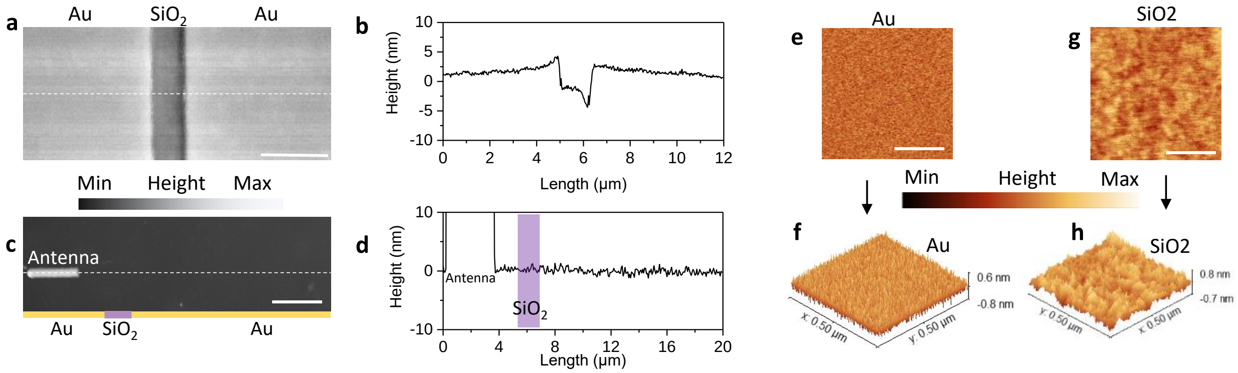

Supplementary Figure 27. Characterization of the Au-SiO_2-Au in-plane sandwich structure by SEM and AFM. (**a**) Topography image of Au-SiO_2-Au in-plane sandwich structure. (**b**) Height profile extracted from a scan along the horizontal dashed line in panel (a). The height of the air gap is ~4 nm. (**c**) Topography image of the Au-SiO_2-Au in-plane sandwich structure covered by graphene/α-MoO_3 and equipped with an antenna. (**d**) Height profile extracted by scanning along the horizontal dashed line in panel (c). **(e-**

h) Characterization of the roughness of the gold (e, f) and SiO_2 (g, h) surface of our samples.

Note that in our sample the Au-SiO_2-Au in-plane sandwich structure is not absolutely flat due to limitations in the fabrication method, which introduces an air gap of less than 5 nm between the α-MoO_3-SiO_2 lens, although such air gap only produces small effects (Supplementary Figures 25-27). In particular, the focusing effect is the result of kinematic constrains (conservation of frequency and parallel wave vector) that should be independent of the presence of a small gap, although the transmitted field amplitude is obviously influenced by it.

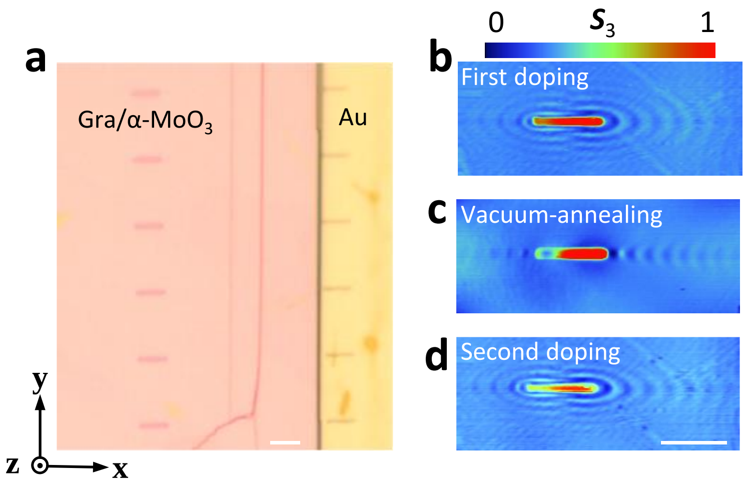

Supplementary Figure 28. Reversibility of gas doping for topological polaritons. (**a**) Optical image of the graphene/α-MoO_3 heterostructures equipped with antennas. (**b**) Infrared nanoimages revealing the reversibility of gas doping for topological polaritons in graphene/α-MoO_3 heterostructures on a 60 nm Au substrate. Near-field images are obtained from the sample in (a) after doping. The graphene Fermi energy is set at E_F=0.7 eV. **(c)** Near-field image obtained from the sample in (b) after applying an annealing treatment. The graphene Fermi energy goes down to E_F=0.3 eV. (**d**) Near-field image from the sample in (c) after re-doping, with the graphene Fermi energy set at E_F=0.7 eV. The incident light wavelength is λ_0 =11.11 μm (900 cm^{-1}) in the experiments. The α-MoO_3 thickness is 240 nm. The scale bars indicate 3 μm. The tuning effect in our gas doping method is reversible because gas molecules are mainly physically adsorbed on the graphene, so that they can strongly attach to or slowly detach from the graphene surface [9-11].

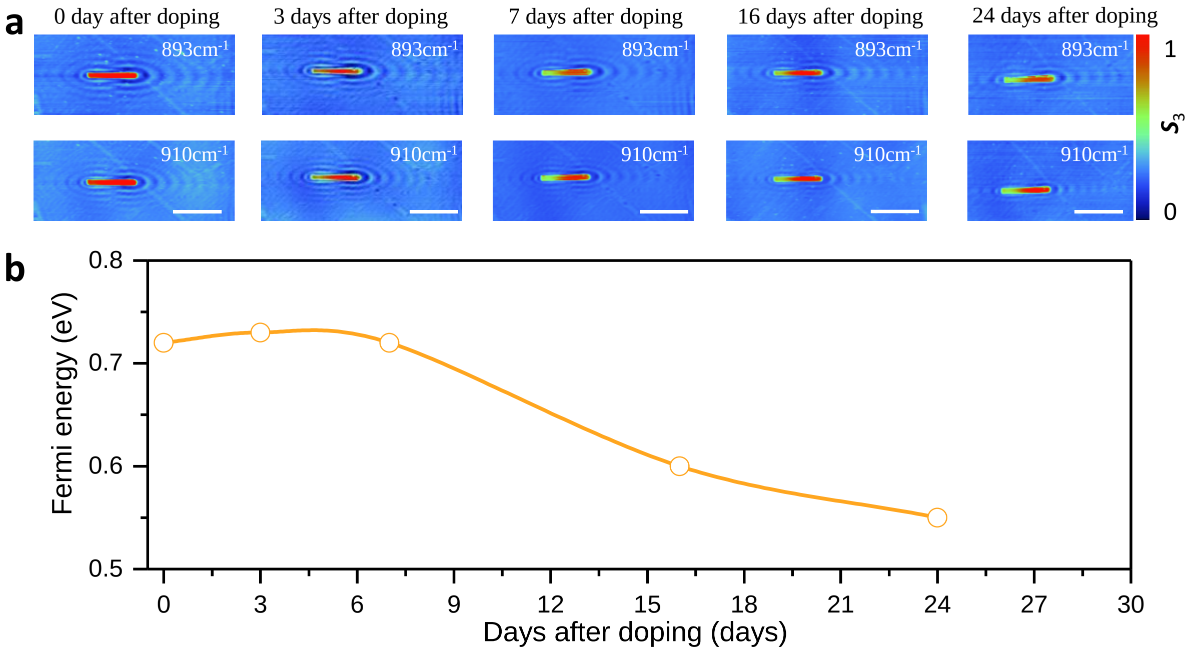

Supplementary Figure 29. Stability of gas doping for topological polaritons. (**a**) Near-field amplitude images revealing the stability of hybrid polaritons by gas doping. The sample is stored for different numbers of days under ambient air conditions. (**b**) Fading of the graphene Fermi energy on α-MoO_3 as a function of time after doping. The slight increase in the graphene Fermi energy after being left for 3 days may relate to water or impurities in the ambient atmosphere. The α-MoO_3 thickness is 240 nm. The scale bars indicate 3 μm. The sample used here to measure the stability of gas doping is the same one as employed to measure reversibility in Supplementary Figure 28.

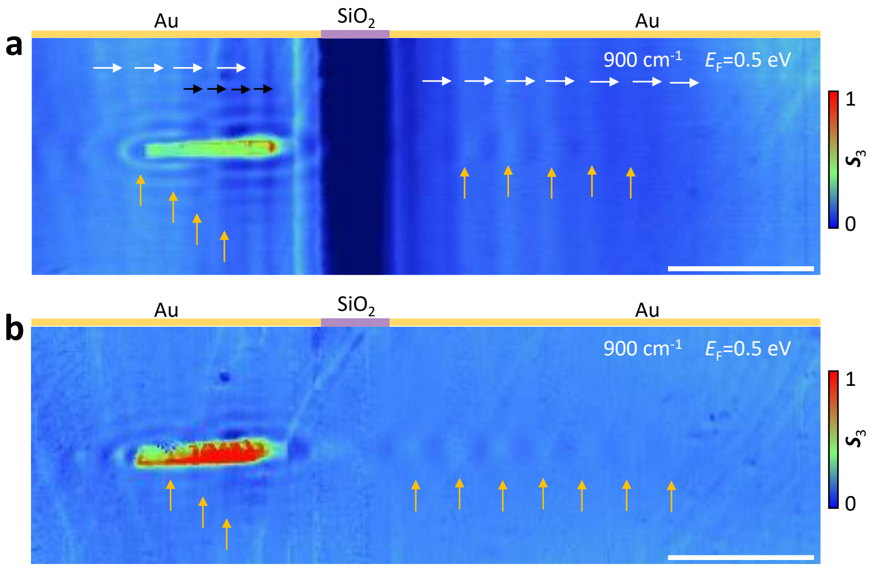

Supplementary Figure 30. Method to extract antenna-launched hybrid polaritons. (**a**) Raw data of the near-field image of a negative refraction lens, as recorded by our s-SNOM setup. The polaritons are launched by a gold antenna (length 3 μm; width 250 nm; thickness 50 nm). The α-MoO_3 is 240 nm thick. The graphene Fermi energy is E_F= 0.5 eV. (**b**) Near-field image of antenna-launched hybrid polaritons corresponding to the raw data in (a), obtained by subtracting the vertical polariton fringes launched or reflected by the gold-SiO_2 interface. The scale bar indicates 3 μm. The horizontal orange and purple stripes on the upper part of the near-field images in (a) and (b) indicate the gold and SiO_2 substrates.

The spot size of the mid-IR beam under the AFM tip is about ~30 μm in lateral size, and therefore, it can cover a large area of the sample in Supplementary Figure 21a, thus leading to tip, antenna, and edge launching of polaritons. Indeed, different types of polariton fringes can be identified. The orange arrows indicate polaritons launched by the antenna. The vertical fringes indicated by the white arrows represent polaritons launched by the gold-SiO_2 interface. The black arrows indicate interference fringes of polaritons launched by the tip and reflected by the gold-SiO_2 interface. The raw data of the near-field image consists of 120×400 pixels (data points), organized in a matrix as:

$$A_0=\begin{bmatrix} a_1^1 & \cdots & a_1^{400} \\ \vdots & \ddots & \vdots \\ a_{120}^1 & \cdots & a_{120}^{400} \end{bmatrix},$$

where the polaritons launched by the antenna occupy the middle part of the image, while in the area at the edge of the image only polaritons launched by the tip and the Au-SiO_2 interface do exist, which can be used as background signals,

$$A_1=\frac{1}{10}\left(\begin{bmatrix} a_1^1 & \cdots & a_1^{400} \\ \vdots & \ddots & \vdots \\ a_5^1 & \cdots & a_5^{400} \end{bmatrix}+\begin{bmatrix} a_{116}^1 & \cdots & a_{116}^{400} \\ \vdots & \ddots & \vdots \\ a_{120}^1 & \cdots & a_{120}^{400} \end{bmatrix}\right).$$

A_1 is obtained from the average value of 10 rows of data from the upper and lower edges to achieve higher accuracy. Therefore, the signal associated with the polaritons launched by the antenna can be obtained from the difference B= A_0-A_1 (Supplementary Figure 30b).

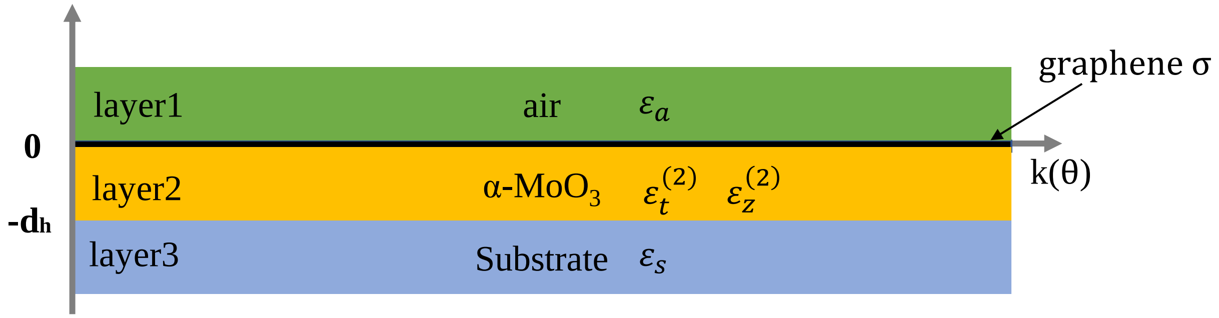

Supplementary Figure 31. Illustration of the geometry considered for the theoretical model. Green, orange, and blue areas stand for different layers. Layer 1 (z>0, air) is a cover layer; layer 2 (0>z>-d_h, graphene/α-MoO_3) is a middle layer; and layer 3 (z<-d_h, SiO_2 or Au) is a substrate. Each layer is described as a dielectric material represented by its dielectric tensor. Monolayer graphene, located on the top of α-MoO_3 at z=0, is described as a zero-thickness current layer characterized by the surface conductivity of this material in the local approximation.

References

1. Zheng Z, *et al*. A mid-infrared biaxial hyperbolic van der Waals crystal. *Sci. Adv*. **5**, eaav8690, (2019).
2. Kischkat, J. *et al*. Mid-infrared optical properties of thin films of aluminum oxide, titanium dioxide, silicon dioxide, aluminum nitride, and silicon nitride. *Appl. Opt*. **51**, 6789-6798, (2012).
3. Babar, S. & Weaver, J. H. Optical constants of Cu, Ag, and Au revisited. *Appl. Opt*. **54**, 477-481, (2015).
4. Gerber, J.A. *et al*. Phase-resolved surface plasmon interferometry of graphene. *Phys. Rev. Lett*. **113**, 055502 (2014).
5. Dai, S. *et al*. Tunable phonon polaritons in atomically thin van der Waals crystals of boron nitride. *Science* **343**, 1125-1129 (2014).
6. Hu, H. *et al*. Active control of micrometer plasmon propagation in suspended graphene. *Nat. Commun*. **13**, 1465 (2022).
7. Woessner, A. *et al*. Highly confined low-loss plasmons in graphene-boron nitride heterostructures. *Nat. Mater*. **14**, 421-425 (2015).
8. Fei Z, Rodin A S, Gannett W, *et al*. Electronic and plasmonic phenomena at graphene grain boundaries. *Nat. Nanotechnol*. **8**, 821-825 (2013).
9. Schedin F, Geim A K, Morozov S V, *et al*. Detection of individual gas molecules adsorbed on graphene. *Nat. Mater*. **6**, 652-655 (2007).

10. Hu H, Yang X, Guo X, *et al*. Gas identification with graphene plasmons. *Nat. Commun*. **10**, 1-7 (2019).
11. Liu H, Liu Y & Zhu D. Chemical doping of graphene. *J. Mater. Chem*. **21**, 3335-3345 (2011).
